# Beyond hours: how conformity-driven overtime influences work withdrawal behavior via dual psychological mechanisms

**DOI:** 10.3389/fpsyg.2025.1711549

**Published:** 2026-01-13

**Authors:** Xin Liu, Xiaochong Wei, Yuhan Wang

**Affiliations:** School of Public Administration and Policy, Renmin University of China, Beijing, China

**Keywords:** conformity, conformity-driven overtime, facades of conformity, overtime work, work withdrawal behavior

## Abstract

**Introduction:**

Despite the prevalence of conformity-driven overtime in East Asian organizations, research on its underlying psychological mechanisms and behavioral consequences remains scarce. Drawing on the Job Demands-Resources model, this study examines how conformity-driven overtime influences work withdrawal behavior through dual pathways—resource depletion and resource compensation—and investigates the moderating role of organizational market orientation.

**Methods:**

A four-wave longitudinal survey was conducted among 943 Chinese employees. Data were analyzed using Structural Equation Modeling to test the hypothesized moderated mediation model.

**Results:**

Results indicated that conformity-driven overtime simultaneously triggered resource depletion via emotional suppression and resource compensation via organization-based self-esteem. Both mechanisms subsequently affected work-related burnout, which in turn predicted work withdrawal behavior. Crucially, organizational market orientation significantly moderated these pathways. In fully market-oriented organizations, where the overtime effort-reward contingency is more predictable and transparent, the detrimental emotional suppression pathway becomes non-significant while the beneficial organization-based self-esteem pathway strengthens, compared to non-market-oriented organizations where overtime evaluative ambiguity prevails.

**Discussion:**

These findings extend JD-R theory by demonstrating that a single job demand—conformity-driven overtime—can elicit distinct, opposing psychological responses (depletion vs. compensation) that are contingent upon the organizational context. Practically, organizations are advised to scrutinize the underlying motives of overtime behavior and mitigate conformity pressures by shifting from a presence-based to a results-oriented performance evaluation system, thereby fostering a healthier work climate.

## 1 Introduction

Overtime work has become a pervasive feature of organizational life across East Asian societies, particularly in China, where rapid economic development, intense labor market competition, and collectivist cultural values have converged to foster a culture of extended working hours (Shen et al., 2025; Hruby, 2018). A substantial proportion of Chinese employees routinely work more than 10 h of overtime per week, with the controversial “996” work schedule—working from 9 a.m. to 9 p.m., 6 days a week—having become normalized across numerous enterprises (Li, 2023). As societal concerns mount regarding the implications of overtime for employee health, family life, and productivity, the phenomenon has sparked considerable debate. While some view overtime as a symbolic marker of diligence and organizational commitment particularly in East Asian working contexts (Babbar and Aspelin, 1998; Geng et al., 2021; Kang et al., 2017), others critique it as symptomatic of exploitative organizational cultures that normalize overwork and reward it at the expense of employee wellbeing (Wang, 2020). This paradox, in which overtime is simultaneously celebrated as a virtue and condemned as a vice, highlights the complex socio-psychological dynamics underlying this organizational phenomenon.

Numerous studies have examined the impact of the duration and/or frequency of overtime work on employees’ behavioral outcomes. Evidence suggests that extended working hours are associated with decreased job satisfaction (Ko and Choi, 2019), reduced motivation to work the following day (Shen et al., 2025), heightened fatigue levels (Beckers, 2008), and increased turnover intentions (Huo and Shi, 2025). However, the vast majority of this literature conceptualizes overtime as a uniform stressor, with predominant attention focused on the impact of how much employees work rather than that of why they work overtime. This quantitative bias has yielded important but incomplete insights: we know that excessive overtime correlates with negative outcomes, yet we understand little about how different driven factors of overtime might fundamentally alter its psychological meaning and behavioral consequences. It has been argued that solely measuring the amount of overtime is insufficient to understand its consequences (Chen et al., 2020). Indeed, different reasons for working overtime may fundamentally shape individuals’ attitude and experience of overtime work; hence its effects (Beckers, 2008; Kim et al., 2022; Wang et al., 2021).

Factors driving overtime work have long attracted attention in the fields of human resource management and organizational psychology (Babbar and Aspelin, 1998; Feldman, 2002; Golden, 2009; Liu et al., 2021; Tan et al., 2023). While existing literature has effectively established that voluntary overtime driven by intrinsic motivation (e.g., self-development, career advancement or fun of work) tends to yield positive outcomes such as increased work engagement and reduced job fatigue (Beckers et al., 2008; Watanabe and Yamauchi, 2018), relatively few studies have examined how contextually nuanced or socially embedded drivers influence employees’ psychological or behavioral outcomes. In particular, conformity—the tendency to align with prevailing social norms and expectations (Cialdini and Goldstein, 2004)—serves as a distinctive and prevalent driver of overtime in China (Peng, 2020) and across many Asian organizational contexts (Ono, 2018; Zhang and Seo, 2016), yet it remains largely overlooked in existing research. This oversight is particularly problematic given that conformity represents a fundamentally different psychological mechanism than other overtime drivers. Distinct from voluntary overtime where behavior aligns with internal volition, and other involuntary forms, such as task-driven or supervisor-mandated overtime, which stem from explicit external demands (Beckers et al., 2008; Tan et al., 2023), conformity-driven overtime (CDO) functions as a behavioral manifestation of facades of conformity (Hewlin, 2003)—specifically, a surface-level alignment with implicit overtime norms to avoid social exclusion, rather than internalized commitment. This behavior emerges from the tension between personal preferences and social expectations, creating a unique form of psychological conflict that is likely to engender distinct behavioral consequences, thus warranting specific investigation. As such, this study aims to address three interrelated questions that speak to the complex and context-sensitive nature of CDO.

First, while conformity behavior is often portrayed as a form of self-silencing that undermines authenticity and psychological wellbeing (Chou et al., 2020; Hewlin, 2009), recent research suggests it may also yield positive outcomes, such as improved social cohesion, perceived social acceptance, and enhanced performance evaluation (Buhr et al., 2021; Jiang and Zhang, 2021). This theoretical tension reflects a fundamental debate in organizational psychology: whether behavioral compliance in the absence of attitudinal alignment necessarily produces negative outcomes, or whether it might serve adaptive functions in certain cultural and organizational contexts. This duality prompts the question: *Does CDO necessarily produce negative effects, or might it also yield compensatory psychological benefits?* Second, although a growing body of research suggests that employees’ conformity in organizational settings can lead to various forms of disengagement behaviors such as cyberloafing (Ye and Qian, 2023) and reduced employee voice (Chou et al., 2020), relatively little is known about the specific consequences of CDO. This oversight is particularly significant given the ambivalent nature of CDO. Since CDO may offer latent positive implications, such as signaling diligence or reinforcing group inclusion, it is theoretically unclear whether such socially validated overtime may also ultimately precipitates disengagement such as work withdrawal behavior (WWB), or if these potential benefits buffer against it. Investigating this specific relationship is critical because it may uncover a productivity paradox inherent in overtime cultures. On the one hand, while CDO is institutionalized as a visible symbol of diligence and viewed by employees themselves as a necessary strategy to demonstrate group fit and avoid exclusion, it may ultimately drive employees—who are unable to physically leave due to normative pressures—to withdraw psychologically as a coping mechanism. Thus, deciphering this relationship is essential to understanding whether the normative pressure to fit in paradoxically fuels the WWB that undermine organizational vitality. On the other hand, given the rising prevalence of disengagement behaviors (e.g., “cyberloafing” or “quiet quitting”) in contemporary workplaces (Hervé and Oh, 2025; Ye and Qian, 2023), identifying and eliminating such hidden antecedents is of paramount importance. Establishing the link between CDO and WWB could offer timely insights for organizations to target the root causes of this present but disengaged phenomenon. As such, this leads to the second question: *Does CDO ultimately contribute to WWB, and if so, through what psychological mechanisms does this occur?* Third, given that CDO may generate double-edged sword effects as we discussed above, a critical question arises: Under what organizational conditions are the negative effects mitigated while the positive effects amplified? Drawing on the Effort-Reward Imbalance framework (Siegrist, 1996) and Control Theory (Rotter, 1954), we posit that the psychological impact of such extra effort (CDO) fundamentally shaped by employees’ perceived control over the effort-reward linkage. In other words, whether employees perceive that their overtime effort is objectively evaluated and predicably rewarded. In light of this theoretical premise, we identify Organizational Market Orientation as the critical moderator for this study. We chose this variable because, within the Chinese institutional context, Organizational Market Orientation structurally shapes organizations’ operational logic of performance management—specifically, whether it is guided by objective and transparent quantitative metrics or dominated by subjective and ambiguous managerial discretion. We posit that this structural variation may fundamentally alter employees’ perceived control over the return on their overtime efforts, thereby moderating the psychological consequences of CDO. Accordingly, we ask: *Do variations in organizations’ market orientation level moderate the psychological consequences resulted by CDO?*

Drawing on recent advances in the Job Demands-Resources (JD-R) model, particularly its recognition of the dual nature of job demands—whereby the same demand can function as either a challenge or hindrance depending on contextual conditions (Bakker’s et al., 2023), we develop a chain mediation model that theorizes CDO as a psychologically ambivalent phenomenon influencing WWB through dual psychological pathways. This dual-pathway conceptualization represents a theoretical innovation: CDO is not uniformly harmful or beneficial, but rather simultaneously triggers resource-depleting via emotional suppression (ES) and resource-generating via organization-based self-esteem (OBSE) processes.

This study makes several contributions to the literature on overtime work, organizational conformity, and human resource management. First, building on prior insights into socially compelled overtime behaviors in East Asian workplaces (Ono, 2018; Watanabe and Yamauchi, 2018), this study further theorizes CDO as a culturally embedded and psychologically ambivalent form of overtime work. Unlike prior research focusing mainly on the quantitative aspects of overtime work, such as frequency and duration (Jiang and Yang, 2025; Yang et al., 2025), we emphasize its qualitative aspects, particularly CDO, providing a new perspective on how overtime can simultaneously generate both negative and positive psychological effects. Our findings suggest that overtime is not a uniform stressor, highlighting the need to examine its underlying drivers to better understand the psychological processes through which overtime work influences employee outcomes. Second, it deepens the application of the JD-R model by developing a dual-pathway model in which the health-impairment process (via ES) and the motivational process (via OBSE) both converge on work-related burnout (WRB) and, in turn, lead to employees’ WWB. More importantly, our findings reveal that although CDO is conceptualized as a job demand, it not only exerts detrimental influences but also fosters employees’ OBSE under certain conditions, thereby exhibiting a dual valence. This empirical evidence substantiates Bakker’s et al. (2023) proposition that job demands may be simultaneously associated with both positive and negative outcomes, thereby extending JD-R model with concrete evidence from the context of overtime work. Third, the study introduces organizational market orientation—a context-specific indicator derived from China’s institutional landscapeeas an institutionally grounded moderator, thereby advancing the contextualization of HRM research in the Chinese setting (Kim and Wright, 2011; Sheldon and Sanders, 2016), and aligns with Demerouti (2025) call to examine how organizational contexts shape employees’ configuration and perception of job demands and resources, illustrating how macro-organizational features can moderate their effects on employee wellbeing. Fourth, it is, to our knowledge, the first study to jointly consider both qualitative (e.g., CDO) and quantitative (e.g., overtime frequency) aspects of overtime in predicting employee behavioral outcomes. Finally, the study offers practical insights by highlighting the psychological and behavioral consequences of CDO. It calls for greater organizational awareness of this overtime phenomenon and for more deliberate, context-sensitive management of overtime work.

## 2 Theory and hypotheses

### 2.1 Conformity and facades of conformity

Conformity is defined in social psychology as the tendency of individuals to adjust their attitudes, beliefs, or behaviors to align with group norms or standards, often as a response to real or perceived social pressure (Cialdini and Goldstein, 2004). A key characteristic of conformity is the normative influence it brings, manifesting in individuals feeling obliged to conform to group norms to obtain social approval, even when they may not agree with the group’s stance (Asch, 1955).

Within organizational contexts, Hewlin (2003) defines facades of conformity as a form of self-presentation where employees suppress their personal values and simulate alignment with organizationally sanctioned norms or values. These facades are constructed through verbal expressions, subtle behavioral cues, gestures, and emotional displays intended to convey value congruence in day-to-day work interactions. The construct is theoretically distinct from impression management (which centers on dyadic influence strategies aimed at specific targets), surface acting (which focuses on emotional display rules in customer-facing roles), and compliance (which involves outward behavioral conformity without consideration of internal authenticity) (Hewlin, 2003).

While initially understood as intentional self-presentational acts, Hewlin (2009) later suggests facades may also emerge as defensive responses to contextual pressures, including non-participative work environments, minority status, and collectivist organizational cultures. Facades may take the form of implicit, habitual expressions—such as dressing in line with organizational expectations or subtly nodding in agreement—especially when employees perceive that expressing divergent values carries social or career-related risks. Specifically, Hewlin (2003, p. 635) characterizes facades as a “survival mechanism,” emphasizing that employees suppress personal values and simulate alignment not to influence others, but to avoid isolation, marginalization, or punishment in rigid organizational environments. This understanding positions facades of conformity as adaptive, even involuntary, responses to real or perceived normative pressure, expanding the construct beyond volitional impression management.

### 2.2 Conformity-driven overtime

In East Asian societies, the cultural valorization of diligence and collective achievement has long shaped prevailing work attitudes (Kang et al., 2017). Hard work is regarded not only as a pathway to individual economic advancement but also as a means of affirming one’s social identity and achieving group recognition (Geng et al., 2021; Ono, 2018). Consequently, in many organizations, remaining at the workplace beyond official hours has transitioned from an occasional necessity to an informal norm that symbolizes commitment and dedication to the collective (Peng, 2020). Over time, this emphasis on relentless effort has contributed to the development of a pervasive culture of overtime work.

However, it is not merely the existence of overtime practices that is notable, but the way in which such practices have been institutionalized as normative prescription within organizations. In East Asian cultural contexts, where the emphasis on collective harmony and relational interdependence is deeply embedded, organizational members are particularly attuned to socially sanctioned behavioral norms (Hom et al., 2025; Triandis, 2001). Over time, this prolonged workplace presence has come to signify not just diligence, but social pressure with implicit standards about what it means to be a responsible organizational member (Peng, 2020). As Cialdini and Trost (1998) explain, social norms function as “rules and standards that are understood by members of a group, and that guide and/or constrain social behavior without the force of laws” (p. 152), often operating through subtle mechanisms of approval, disapproval, or anticipated social evaluation. These norms are not merely abstract rules, but shared behavioral expectations embedded within the social fabric of organizational life. At first, individuals often perceive these norms through descriptive cues (Fishbein and Ajzen, 2011)—that is, by observing what others routinely do or avoid in the workplace (Rivis and Sheeran, 2003). Over time, repeated exposure to such implicit behavioral patterns shapes individual perceptions of which behaviors are appropriate, acceptable, or deviant in the workplace (Dose, 1997), corresponding to what Fishbein and Ajzen (2011) define as “injunctive norm”—what ought to be done. Consequently, employees begin to cognitively incorporate such organizational values and norms into their evaluative standards, not necessarily as internalized beliefs, but as practical guides for navigating daily work behavior (Dose, 1997). It is interesting to note that such behavioral compliance may often be reinforced by embedded evaluative mechanisms, such as informal rewards for compliance and subtle disincentives for deviation (Hom et al., 2025). More importantly, these tacit evaluative standards, once cognitively incorporated, may not only shape individual self-regulation, but also function as implicit benchmarks for assessing the conformity of others within the organizational environment (Enz, 1988; Frink and Klimoski, 1998). As a result, Tetlock et al. (1989) argue that under conditions of accountability, where individuals anticipate judgment from others, conformity serves as a cognitively efficient coping strategy. Rather than generating authentic justifications under uncertainty, individuals tend to conform to prevailing expectations to avoid negative evaluations and maintain social order. In this way, normative prescriptions become embedded in daily organizational life not just as behavioral cues, but also as systems of felt accountability that regulate individual conduct (Hom et al., 2025) and serve as shared standards of appraisal for evaluating others (Hewlin, 2009).

As such, in the context of overtime work, the practice of staying late itself has become a subtle evaluative standard—one that exerts pervasive social pressure on employees to conform, even in the absence of explicit mandates (Kim et al., 2022). Given the inherently demanding nature of overtime, particularly involuntary overtime, which often involves extended physical and psychological labor (Beckers et al., 2008), its institutionalization as a normative expectation inarguably amplifies the psychological burden on employees. Recent qualitative studies have documented how overtime has shifted from a voluntary act of diligence to a socially compelled behavior. Peng (2020) observed that white-collar employees in Shanghai frequently experienced psychological conflict when deciding whether to leave work on time, primarily due to concerns about negative evaluations from supervisors and colleagues. Beyond personal perceptions, Wang (2020) further revealed that managerial controls, strategically reinforced by cultural expectations, have been deliberately implemented to institutionalize excessive working hours and embed conformity into daily organizational routines. These formal-informal management mechanisms show how normative pressure is operationalized through organizational structure. Moreover, Kang et al. (2017) illuminate the motivational base of such conformity by identifying deep-rooted Confucian ideals, such as deference to authority and prioritization of collective harmony, as key cultural drivers that lead employees to accept and even rationalize prolonged overtime, despite potential conflicts with their personal interests and wellbeing. This phenomenon is particularly pronounced in China, where the absence of institutional work-life protections, combined with intensifying work demands and the enduring influence of Confucian values, has collectively normalized long working hours as an accepted, even expected, feature of professional life (Xiao and Cooke, 2012).

In such environments, employees’ decisions to remain at work beyond official hours are often less a reflection of intrinsic motivation or actual task necessity than a response to pervasive normative pressures. Although externally exhibiting signs of commitment and diligence, employees may internally experience ambivalence or even resistance toward the overtime culture. This overtime pattern, characterized by a surface-level alignment with organizational expectations despite underlying divergence in personal attitudes, can be understood as Conformity-driven overtime (CDO). Conceptually, such behaviors represent a tangible manifestation of facades of conformity (Hewlin, 2003, 2009), wherein employees suppress authentic preferences to simulate alignment with organizational values, particularly in contexts where visible conformity carries significant social and evaluative weight. While some related conceptions such as impression management, surface acting, and compliance could also describe phenomena with overlapping behavioral features like CDO, they do not offer the precise theoretical lens required to understand the specific psychological dynamics of CDO. First, unlike impression management, which is typically a proactive and strategic tactic aimed at acquiring rewards (Bolino, 1999), CDO functions primarily as a defensive response—a “survival mechanism”—to maintain social belonging and avoid marginalization. Second, distinct from surface acting, which focuses on the momentary regulation of emotional displays (e.g., smiling) in service interactions (Grandey, 2003), CDO involves behavioral/temporal conformity—remaining physically present at work—rooted in broader value incongruence rather than specific display rules. Third, unlike compliance which implies obedience to explicit supervisory mandates, CDO arises from implicit normative pressures where employees feel compelled to conform even in the absence of direct orders. Thus, the facades of conformity framework best capture the internal value tension and defensive nature inherent in CDO. Given the underlying misalignment between internal attitudes and external behaviors, CDO is likely to engender distinct psychological experiences and work-related outcomes, which will be delineated in the following sections.

### 2.3 Job demands-resources model

To understand how CDO may affect employee outcomes, this study adopts the JD-R model as its primary theoretical framework. The JD-R model posits that job characteristics can be broadly categorized as either job demands—aspects of work that require sustained effort and are therefore associated with both physical and emotional costs—or job resources, which help individuals meet work goals, foster learning and development, and mitigate the impact of demands (Demerouti et al., 2001). Crucially, these characteristics instigate two distinct psychological processes: the health-impairment process, where high job demands deplete employees’ mental and physical resources leading to strain (e.g., burnout), and the motivational process, where job resources foster engagement and personal growth. Furthermore, these two forces do not operate in isolation. The interplay between high demands and insufficient resources most strongly predicts strain-related outcomes, whereas abundant resources can buffer the adverse effects of demands, promoting resilience even under pressure (Bakker et al., 2005).

While traditional JD-R research often dichotomizes demands as either “challenge,” which purely exert resource-enhancing outcomes, or “hindrance” which solely result in resource-depleting effects (Crawford et al., 2010), recent theoretical advancements explicitly posit that the same job demand can simultaneously trigger both positive and negative outcomes depending on its appraisal (Bakker’s et al., 2023). In terms of our research context, CDO constitutes a significant job demand requiring sustained effort. However, drawing on this updated perspective, we conceptualize CDO not as a static hindrance or challenge, but as a hybrid demand characterized by psychological ambivalence that may result in both resource-enhancing and resource-depleting effects. Accordingly, we map the dual effects of CDO directly onto the two core processes (Health-impairment and Motivational) of the JD-R model.

On the one hand, consistent with the health-impairment process, the normative pressure of CDO acts as a hindrance. The perceived conflict between personal preference and social expectation creates a psychological burden that depletes energy, thereby fueling strain-related outcomes. On the other hand, consistent with the motivational process, the symbolic value of CDO acts as a challenge-like aspect. By signaling diligence and securing social inclusion, successful conformity generates compensatory psychological resources, which can buffer the strain of demands. These potential dual pathways are detailed and operationalized in the following sections.

### 2.4 Conformity-driven overtime and work withdrawal behavior

Work withdrawal behavior (WWB) refers to a set of psychological and behavioral disengagement responses that employees exhibit when they are dissatisfied with their work environment but are unwilling or unable to leave the organization. The concept has been extensively discussed in organizational psychology literature. Hanisch and Hulin (1990) defined work withdrawal as a “retreat behavior” aimed at reducing one’s exposure to undesirable work conditions while still maintaining formal organizational membership. Similarly, Lehman and Simpson (1992) conceptualized withdrawal as a form of “cold coping strategy” used by employees to avoid emotional strain or conflict while preserving employment status. This construct is often distinguished from turnover behavior in that while both emerge from discontent, withdrawal represents a compromise between remaining and exiting. As Harrison et al. (2006) noted, withdrawal behavior encompasses a range of actions including lateness, absenteeism, and reduced effort, and is more prevalent than actual turnover because it allows employees to avoid the economic, social, and relational costs of quitting.

While facades of conformity—employees’ deliberate misalignment of private values and public behavior—has been empirically linked to turnover intention (Hewlin, 2009), we contend that turnover is neither the most immediate nor the most common behavioral consequence of prolonged exposure to normative pressure. Drawing from Hom et al. (2025), who emphasized the extensive relational, familial, economic, and career-related costs that constrain voluntary quitting, we argue that employees are more likely to remain in their positions despite strong psychological conflict. This is especially likely when conformity does not stem from internalization of organizational norms but is instead a skin-deep response enacted to avoid social disapproval (Hewlin, 2009). In such double-bind situations—where employees are neither willing to fully conform nor inclined to leave—WWB may emerge as a rational, resource-conserving alternative. The Conservation of Resources (COR) theory (Hobfoll, 1989) posits that individuals strive to preserve emotional and psychological resources in the face of sustained demands. Thus, work withdrawal behaviors such as reduced involvement, minimal effort, or emotional detachment are likely to serve as mechanisms to avoid deeper resource depletion.

This theoretical logic is supported by multiple empirical studies. For instance, Scott and Barnes (2011), using a multilevel field study of bus drivers, demonstrated that surface acting—a form of emotional labor that parallels superficial conformity—predicts daily work withdrawal through increased negative affect. Similarly, Peng and Li (2023) found that emotional exhaustion mediated the relationship between surface acting and work withdrawal among hospitality employees in China, reinforcing the idea that behavioral compliance under emotional strain prompts disengagement. Wessel et al. (2020) provided further evidence by showing that lack of role authenticity—defined as the inability to enact one’s work role in alignment with one’s true self—significantly predicted work withdrawal over time. Notably, these findings are echoed by Stengård et al. (2016), who showed that employees who felt “locked-in” to unsatisfactory jobs but perceive few alternatives, due to labor market conditions or personal constraints, usually report lower engagement and higher withdrawal behaviors across a longitudinal period. These findings collectively indicate that when employees are pressured to conform but lack the freedom to leave, withdrawal behavior may become a psychologically efficient form of resistance.

CDO, as previously discussed, often entails outward compliance with organizational norms in the absence of genuine alignment or desire, making it psychologically costly over time. Given the risks and constraints associated with turnover, employees are more likely to adopt disengagement strategies that allow them to cope while remaining formally employed. We therefore propose the following hypothesis:

*H1:* CDO is positively associated with WWB.

### 2.5 Work-related burnout

Burnout is a psychological syndrome that develops in response to prolonged exposure to emotionally taxing work conditions and is widely recognized as a central indicator of occupational strain (Maslach et al., 2001). Traditionally, burnout has been conceptualized through the Maslach Burnout Inventory (MBI), which identifies three core dimensions: emotional exhaustion, depersonalization (or cynicism), and reduced personal accomplishment. Among these, emotional exhaustion—the sense of being emotionally overextended and depleted—is generally regarded as the core component (Leiter and Maslach, 2016).

However, in this study we adopt the Copenhagen Burnout Inventory (CBI) developed by Kristensen et al. (2005), which offers a complementary conceptual framework. The CBI conceptualizes burnout not as a unitary syndrome, but as a set of fatigue experiences that differ based on their attributed source. It thus divides burnout into three distinct yet interrelated domains: personal burnout, work-related burnout, and client-related burnout. As the authors emphasize, “the three scales can be used independently in accordance with the populations being studied and the theoretical questions being elucidated” (Kristensen et al., 2005, p. 205). This makes the CBI particularly suitable for studies that seek to isolate specific contextual sources of burnout. Given that this study focuses on employees’ psychological responses to CDO, rather than general life fatigue or exhaustion derived from interactions with clients or service recipients, we therefore focus specifically on the WRB dimension (The degree of physical and psychological fatigue and exhaustion that is perceived by the person as related to his/her work, p. 197) proposed in the Copenhagen Burnout Inventory (CBI). This construct captures the extent to which employees experience emotional and physical exhaustion that they attribute specifically to their work tasks, conditions, and organizational environment (Kristensen et al., 2005), making it theoretically consistent with the nature of our research context.

Despite extensive research on the link between overtime work and burnout, findings remain inconclusive (Rabenu and Aharoni-Goldenberg, 2017). Most studies have examined burnout in relation to the length and frequency of overtime work (Jiang and Yang, 2025), paying less attention to the underlying reasons driving employees to work overtime. Some research suggests a direct relationship between extended work hours and burnout. For example, Rupert et al. (2013) found a significant positive correlation between weekly working hours and mental exhaustion. Similarly, limiting work hours has been shown to reduce burnout (Martini et al., 2006). However, other studies challenge the assumption that longer hours alone lead to burnout. Shirom et al. (2010) found no direct correlation between total work hours and burnout, arguing instead that burnout is more strongly influenced by perceived workload increases and reduced job autonomy. Likewise, Schaufeli et al. (2008) reported no clear link between overtime hours and managers’ burnout.

This inconsistency in the literature underscores the need to move beyond a purely quantitative approach to studying overtime (Chen et al., 2020). A more nuanced understanding of how WRB arises from CDO and contributes to WWB is therefore essential.

### 2.6 Mediating role of emotional suppression in the relationship between conformity-driven overtime and work-related burnout

Emotional suppression (ES) is a response-focused emotion regulation strategy in which individuals inhibit the outward expression of internally experienced emotions after those emotions have already been activated (Gross, 1998; Gross and John, 2003). Unlike antecedent-focused strategies such as cognitive reappraisal, suppression occurs later in the emotion-generative process and reflects a chronic tendency to conceal affective authenticity to manage interpersonal impressions (Gross and John, 2003).

It is also important to distinguish ES from surface acting, as the two are often conflated in workplace emotion regulation research. Surface acting typically involves the outward display of unfelt positive emotions, primarily to comply with formal emotional display rules in service-related roles (Grandey, 2003; Hülsheger and Schewe, 2011). In contrast, ES entails withholding the expression of already felt negative emotions, particularly in social contexts where individuals feel compelled to conform to implicit group norms in order to avoid interpersonal tension or social disapproval (Hewlin, 2009; Cialdini and Trost, 1998). Given these distinctions, ES may emerge as a more salient regulatory response compared to surface acting especially in the context of CDO. Unlike service contexts where employees must project cheerfulness, CDO occurs in internal team environments marked by implicit normative expectations. As such, in these settings, suppression reflects emotional self-censorship under social pressure: employees remain physically present while concealing inner discontent and outwardly complying without overt resistance.

A substantial body of research has shown that ES is widely regarded as a maladaptive strategy that has been empirically linked to psychological distress, emotional exhaustion, and reduced authenticity in relationships (Butler et al., 2003; Grandey, 2003; Moore et al., 2008). Within the JD-R model, CDO fosters a climate that implicitly discourages emotional authenticity, thereby reinforcing suppressive behavior. We position this dynamic within the health-impairment process of JD-R model: ES functions as an emotionally taxing regulatory burden that depletes internal resources while offering little intrinsic return. Over time, this cumulative resource loss contributes to strain-related outcomes, specifically WRB. Accordingly, this study proposes that:

*H2a:* CDO is positively associated with ES.

*H2b:* ES is positively associated with WRB.

*H2c:* ES plays a mediating role in the Relationship between CDO and WRB.

### 2.7 Mediating role of organization-based self-esteem in the relationship between conformity-driven overtime and work-related burnout

Organization-based self-esteem (OBSE) refers to the degree to which organizational members believe they are capable, significant, and worthy within their employing organization (Pierce et al., 1989). This construct reflects a self-evaluative perception of being a valued contributor in one’s organizational role—one that integrates both competence and belonging, shaped through workplace experiences.

More importantly, OBSE is not a stable trait but a context-sensitive construct, developed through social interactions and structural feedback embedded in the organizational environment (Pierce and Gardner, 2004). Compared with related constructs such as global self-esteem (a generalized sense of self-worth) and task-specific self-esteem (competence tied to specific tasks), OBSE uniquely captures individuals perceived social worth within their organization. Pierce et al. (1989) emphasized that OBSE is especially predictive of organizationally embedded outcomes such as job satisfaction, organizational commitment, citizenship behavior, and performance, thereby offering a more precise lens for understanding employee responses to organizational conditions.

Although often framed as compliance or loss of authenticity, recent scholarship suggests that conformity can also play a constructive role in how individuals adapt to organizational environments. At the organizational level, Hanaki and Owan (2013) demonstrate that in complex or uncertain task environments, high-conformity groups outperform high-autonomy ones over time. This advantage arises because conformity fosters cognitive alignment, behavioral coordination, and normative clarity, which together enhance organizational learning and collective performance. Similarly, Buhr et al. (2021) show that moderate conformity can strengthen an organization’s perceived authenticity and legitimacy, particularly when external evaluators rely on categorical norms to assess credibility. These findings suggest that conformity may not simply reflect social pressure but can operate as a productive force in organizational systems.

At the individual level, conformity can also serve psychologically adaptive functions. When individuals operate under conditions of accountability—where they anticipate being evaluated by others—conforming to expectations offers a cognitively efficient coping strategy that reduces the burden of generating personal justifications in ambiguous or uncertain situations (Tetlock et al., 1989). Rather than stemming solely from fear of sanctions, conformity often reflects a proactive means of managing interpersonal impressions and sustaining social harmony. Consistent with this micro-level view, Cialdini and Trost (1998) emphasize that social norms exert influence not only through the threat of disapproval but also through the promise of social acceptance, approval, and belonging. These normative rewards further incentivize individuals to align their behavior with group expectations, especially in contexts where interpersonal harmony and group cohesion are valued. For example, empirical research by Jiang and Zhang (2021) has highlighted that employees who experienced workplace ostracism frequently adopted facades of conformity as a protective response to avoid further exclusion. Importantly, such behaviors were not merely tolerated but consequently were even rewarded with favorable performance evaluations, particularly when supervisors themselves endorsed collectivist values. These findings suggest that even defensive or strategic forms of conformity may be positively sanctioned under culturally shared expectations.

In the context of CDO, we argue that such behavior, though rooted in normative expectations, may function as a mechanism for social validation. Overtime is often imbued with moral connotations and interpreted as a marker of diligence, loyalty, team commitment, and role identity within the organization particularly in East Asian work environments (Babbar and Aspelin, 1998; Geng et al., 2021; Kang et al., 2017). Building on these cultural interpretations and considering that normative compliance is often socially rewarded (Hom et al., 2025; Fishbein and Ajzen, 1975), employees who engage in CDO may receive implicit acknowledgment or positive reinforcement from peers and supervisors for demonstrating compliance with informal behavioral norms. According to Pierce and Gardner (2004), OBSE is shaped through the individual’s interpretation of organizational feedback regarding their role-consistent behaviors. When employees perceive that their compliance with informal behavioral norms (e.g., staying late) is socially valued, such perceived approval may become internalized, reinforcing their belief that they are respected and important members of the organization.

OBSE has been widely associated with a range of positive psychological and behavioral outcomes in the workplace. As a self-evaluative belief grounded in perceived organizational appreciation and role validation, OBSE enhances employees’ sense of competence, belonging, and self-worth (Pierce and Gardner, 2004). Empirical studies demonstrate that higher OBSE is linked to lower emotional exhaustion and reduced turnover intentions (Bowling et al., 2010), and can mediate the relationship between supportive work climates and lower burnout levels (Yuan et al., 2024). Building on these findings, this study draws on the JD-R model to position these effects within the motivational process. We conceptualize OBSE as a compensatory psychological resource generated by the social validation of CDO, which serves to buffer the strain associated with normative pressure. Accordingly, this study proposes that:

*H3a:* CDO is positively associated with employees’ OBSE.

*H3b:* OBSE is negatively associated with WRB.

*H3c:* OBSE plays a mediating role in the relationship between CDO and WRB.

### 2.8 Work-related burnout and work withdrawal behavior

Work-related burnout (WRB), as conceptualized by the Copenhagen Burnout Inventory (CBI), refers to the experience of persistent physical and emotional exhaustion attributable to one’s job responsibilities (Kristensen et al., 2005). This form of burnout emphasizes general fatigue arising from prolonged work strain and is distinct from more relational (e.g., client-related) or dispositional (e.g., personal) forms. As such, it provides a context-sensitive lens for understanding employees’ affective responses to job-specific pressures.

Within the broader stress-strain framework, WRB is widely regarded as a proximal antecedent of employee withdrawal behavior. Conservation of Resources (COR) theory (Hobfoll, 1989) suggests that individuals experiencing emotional exhaustion engage in withdrawal to preserve remaining psychological resources. These withdrawal responses may take the form of psychological detachment, reduced task engagement, or avoidance of effort-intensive work interactions. Empirical findings support this perspective. Using longitudinal data and the CBI, Borritz et al. (2006) demonstrated that employees reporting higher WRB were significantly more likely to take long-term sickness absence. Similarly, Lee et al. (2023) found that emotional exhaustion was a key predictor of unplanned absenteeism among healthcare workers during the COVID-19 pandemic. Gaspar et al. (2024) further emphasized that work-induced burnout contributes to both emotional disengagement and cognitive distancing, precursors to broader withdrawal patterns. These findings converge to support the view that WRB may serve as an immediate driver of WWB. Accordingly, this study hypothesizes that:

*H4:* WRB is positively associated with WWB.

### 2.9 Chain mediating role of emotional suppression/organization-based self-esteem and work-related burnout in the relationship between conformity-driven overtime and work withdrawal behavior

Building on the above discussion, this section integrates the indirect pathways through which CDO exerts its influence on employees’ WWB. While the direct relationship between CDO and WWB reflects the broader motivational and emotional consequences of involuntary overwork, a more nuanced understanding requires tracing how this effect unfolds through successive psychological mechanisms. Based on our theoretical framework, we propose that two distinct chain-mediating processes underlie this relationship.

The first pathway reflects a resource-depleting mechanism. It begins with the emotional strain likely triggered by CDO, as employees suppress their authentic emotional responses to conform to organizational expectations. Over time, this ES may generate accumulating psychological pressure, which could eventually manifest as WRB. WRB, in turn, may prompt WWB as employees attempt to conserve their remaining psychological resources.

The second pathway highlights a resource-compensatory mechanism. CDO, although externally imposed, may enhance employees’ sense of being valued and accepted by the organization, thereby elevating their OBSE. As a form of positive self-evaluation rooted in one’s organizational membership, OBSE may function as a psychological resource that buffers against emotional exhaustion. Employees with higher OBSE are more likely to perceive their efforts as meaningful and recognized, which may help mitigate the onset of WRB. In turn, lower levels of burnout could be associated with reduced WWB. Together, these two pathways articulate how both psychological demands and resources initiated by CDO converge onto WRB, which serves as a pivotal driver of WWB. To empirically capture this dual-chain process, we propose the following hypotheses:

*H5:* ES and WRB sequentially mediate the relationship between CDO and WWB. Specifically, CDO exhibit a negative correlation with ES, while ES demonstrates a positive correlation with WRB, which, in turn, is positively correlated with WWB.

*H6:* OBSE and WRB sequentially mediate the relationship between CDO and WWB. Specifically, CDO exhibit a positive correlation with OBSE, while OBSE demonstrates a negative correlation with WRB, which, in turn, is positively correlated with WWB.

### 2.10 The moderating role of the organizational market orientation level

In organizational behavior research, a foundational assumption is that employee perceptions and reactions to organizational demands are inseparable from the broader organizational contexts in which they are situated (Mischel, 1977; Meyer et al., 2010). As Johns (2006) emphasized in his influential critique on the “forgotten context,” employees’ cognitive, affective, and behavioral responses to organizational demands cannot be fully understood without accounting for the contextual variables (e.g., organizational policies, governance structures, and performance evaluative systems), which fundamentally shape how individuals interpret and act upon them.

Building on this perspective, we seek to identify the boundary conditions that regulate the dual pathways of CDO. In specific, the conditions under which the resource-compensating effect (via OBSE) is amplified while the resource-depleting effect (via ES) is mitigated. According to the Effort-Reward Imbalance framework (Siegrist, 1996), the pathogenic impact of high-effort demands is exacerbated when rewards are uncertain. Critically, this uncertainty tends to be more damaging when outcomes are perceived to be shaped by external factors, such as hierarchical powerholders rather than personal effort (Spector, 1982). Such dependency undermines the clarity of the effort–reward contingency and diminishes individual perceived control (Rotter, 1954), thereby intensifying the strain of employees’ extra effort. As such, we posit that the psychological valence of CDO depends fundamentally on employees’ perceived control over their overtime-reward linkage. To capture the structural determinants of this effort-reward contingency within the Chinese institutional context, we introduce Organizational Market Orientation as the focal moderator. Specifically, drawing on the official classification framework outlined in the “*Guiding Opinions of the Central Committee of the Communist Party of China and the State Council on Deepening the Reform of State-Owned Enterprises*” *(Zhongfa [2015] No. 22)*, we apply this classification scheme to organizations included in our empirical sample, categorizing them into three types—fully market-oriented (e.g., private enterprises or commercially operated SOEs in market-oriented sectors), semi-market-oriented (e.g., state-owned enterprises that seek profitability while simultaneously undertaking strategic and policy responsibilities mandated by the state) (Huang et al., 2024; Lin et al., 2020), and non-market-oriented (e.g., public service institutions and administrative units delivering non-profit public goods and service)—based on the degree of market competition they face and the strength of profit-seeking imperatives embedded in their operational logic. The theoretical rationale for how these varying levels of market orientation systematically shape employees’ appraisal of CDO and its subsequent psychological outcomes is detailed in the following paragraphs.

The organizational market orientation categories introduced above differ not only in their exposure to market forces and profit imperatives, but also in how they manage and evaluate employee performance. A particularly salient institutional distinction concerns the design of performance management systems, as public and private sector organizations vary substantially in their goal structures, evaluative criteria, and accountability mechanisms (Rainey, 2009; Van Helden and Reichard, 2016). Public organizations, typically characterized by low levels of market orientation, governed predominantly by politically constituted hierarchies—tend to operate under goal structures that are highly multiple, vague, and conflicting compared to the goals of business firms (Chun and Rainey, 2005; Van Helden and Reichard, 2016). These goal-related ambiguities, coupled with the rigid and rule-bound organizational systems of public organizations (Boyne, 2002), often make it more difficult for them to implement performance management systems in a coherent and effective manner (Virani and van der Wal, 2023). Meanwhile, individuals’ performance standards in public organizations tend to be diffuse, and appraisal practices often rely heavily on subjective managerial discretion (Perry and Rainey, 1988; Rainey, 2009). This subjectivity is further reinforced by the nature of civil service work itself, as many public-sector jobs involve tasks that are hard to quantify and encompass diverse responsibilities, which makes the use of objective performance indicators for attributing individual contributions inherently challenging (Olken and Pande, 2013; Finan et al., 2015). As a result, individuals in public organizations tend to perceive limited alignment between job performance and concrete rewards such as promotion or pay increases (Rainey, 1983). In contrast, private enterprises, as prototypical market-driven entities subject to decentralized control via market mechanisms, are typically guided by clearer, quantifiable objectives and cost-efficiency imperatives, fostering performance systems that dramatically emphasize measurable outputs and individualized merit-based rewards (Perry and Rainey, 1988; Solomon, 1986; Van Helden and Reichard, 2016). These arrangements may facilitate consistent and outcome-based evaluations, thereby reinforcing employees’ perceptions of a transparent effort–reward linkage.

In public organizations where evaluative standards usually prioritize hierarchical conformity over measurable merit (Meyer, 1979), the link between effort and reward becomes increasingly mediated by interpersonal factors, especially supervisors’ subjective preferences (De Janvry et al., 2023). This structural ambiguity is particularly evident in the evaluation of overtime work, which, as a form of visible extra effort, often faces substantial evaluative uncertainty in subjective appraisal systems. Guo et al. (2021), in an empirical study of Chinese workplaces, found that the performance impact of overtime work is contingent upon supervisors’ affective preferences. For example, when supervisors hold unfavorable attitudes toward an employee, overtime is more likely to be interpreted as ingratiation (i.e., exemplification), thereby reducing performance ratings. In contrast, when supervisors view the employee favorably, overtime is more often attributed to conscientiousness—yet such positive attributions do not necessarily yield higher performance evaluations. These findings illustrate that when performance evaluation is dominated by managerial discretion, the extent to which overtime effort translates into positive performance evaluation is highly unpredictable. The same overtime work behavior may be interpreted differently depending on supervisors’ subjective preferences, reinforcing the evaluative uncertainty faced by employees.

This evaluative uncertainty, particularly when effort-related outcomes are perceived to be significantly shaped by external factors such as hierarchical powerholders rather than personal effort, could undermine individuals’ perception on the clarity of the effort–reward contingency (Siegrist, 1996; Siegrist et al., 2004; Spreitzer, 1995) and consequently lead to diminished perceived control (Parker, 1993; Spector, 1982). Perceived control, defined as the belief that one’s own effort will lead to desired outcomes (Rotter, 1954), implies that individuals see themselves as active agents in shaping their environment, rather than as passive recipients of arbitrary decisions (Ajzen, 2002). This sense of control has critical implications for individual experience, such that individuals’ motivational and affective functioning depends on the extent to which they believe that their effort will lead to predictable and meaningful outcomes (Hervé and Oh, 2025).

On the one hand, when this sense of contingency is weakened and employees perceive little control over whether their extra efforts will be rewarded, they are likely to engage in ES as a coping mechanism. According to Gross (2014) and Sheppes et al. (2014), individuals tend to choose suppression over open expression when they anticipate that emotional expression will be ineffective or socially costly. In organizational contexts where performance outcomes are predominately influenced by opaque evaluative standards or hierarchical discretion, employees may judge that voicing frustration is unlikely to improve their standing—and may, in fact, jeopardize it and exhibit compliance (Spreitzer, 1995; Morrison and Milliken, 2000). Consequently, ES becomes not merely a passive response, but a strategic form of self-regulation aimed at avoiding interpersonal conflict and preserving a facade of professionalism (Hewlin, 2003, 2009).

On the other hand, perceived control is also central to how employees evaluate OBSE within the organization. As Pierce et al. (1989) defined, OBSE is the degree to which individuals believe they are capable, significant, and valuable within their organizational context. When individuals perceive that their actions exert little influence over valued outcomes, they tend to see themselves as less empowered, competent, and impactful contributors within their organizations (Filstad et al., 2019; Lee et al., 2018). Moreover, as noted by Semmer et al. (2007), effort–reward imbalance is not only a source of perceived unfairness, but also a symbolic threat to one’s identity as a capable contributor. Similarly, Kinman and Jones (2008) found that chronic exposure to such imbalances undermines employees’ perceived self-worth and psychological wellbeing. Such ambiguity in the effort–reward linkage may lead employees to question whether their efforts and contributions are acknowledged at all. This uncertainty disrupts the feedback mechanisms necessary for sustaining OBSE, which depends on consistent recognition of one’s value. Indeed, OBSE is not solely an internal belief, but a social construction shaped by evaluative cues from others (Pierce and Gardner, 2004). Although OBSE has often been linked to structural job features such as autonomy and participative decision-making (Gardner, 2020; Norman et al., 2015), it is ultimately grounded in employees’ subjective experience of being recognized and effective in their roles (Pierce and Gardner, 2004). Thus, when the effort–reward linkage becomes ambiguous and employees experience diminished perceived control, their OBSE is likely to decline.

Taken together, we propose that the psychological impact of CDO, particularly its influence on ES and OBSE, is closely tied to employees’ perceived control over whether their overtime efforts will be fairly recognized and rewarded. Specifically, when employees engage in CDO within organizational settings where performance appraisal is marked by subjectivity, ambiguity, and substantial dependence on managerial discretion, they are more likely to perceive a disconnect between their overtime efforts and potential rewards, thereby undermining perceived control. As prior sections have shown, diminished perceived control may increase employees’ reliance on ES as a defensive regulation strategy, while simultaneously eroding their sense of OBSE due to a perceived lack of efficacy and recognition. By contrast, organizations that implement structured and transparent performance evaluative systems may mitigate the psychological burden of CDO. When employees engage in CDO within organizational settings where performance appraisal is governed by clear, consistent, and objective standards such as the use of quantifiable performance metrics, documented overtime management and compensation policies (e.g., overtime pay or compensatory leave) and multi-source feedback systems, their efforts—especially overtime work—is more likely to be objectively tracked, fairly evaluated, and appropriately rewarded. These conditions reduce employees’ ambiguity in the effort–reward linkage and strengthen perceived control, which may function as a job resource within the JD–R framework. In turn, this job resource could buffer the strain associated with CDO, leading to less emotional dissonance and a weaker detrimental effect on employees’ OBSE. This proposition aligns with prior research emphasizing the importance of appraisal consistency and institutional clarity in shaping employees’ psychological responses to workplace demands (Meyer et al., 2010; Rainey, 2009).

Based on above discussion, we contend that the higher an organization’s market orientation level, the more likely employees’ overtime efforts are to yield predictable performance returns, thereby attenuating the negative psychological effects of CDO. Accordingly, we hypothesize the following:

*H7a.* The positive relationship between CDO and ES is hypothesized to be weaker in fully market-oriented organizations than in semi-market-oriented organizations.

*H7b.* The positive relationship between CDO and ES is hypothesized to be weaker in fully market-oriented organizations than in non-market-oriented organizations.

*H7c.* The positive relationship between CDO and ES is hypothesized to be weaker in semi-market-oriented organizations than in non-market-oriented organizations.

*H8a*. The positive relationship between CDO and OBSE is hypothesized to be stronger in fully market-oriented organizations than in semi-market-oriented organizations.

*H8b.* The positive relationship between CDO and OBSE is hypothesized to be stronger in fully market-oriented organizations than in non-market-oriented organizations.

*H8c.* The positive relationship between CDO and OBSE is hypothesized to be stronger in semi-market-oriented organizations than in non-market-oriented organizations.

**FIGURE 1:**
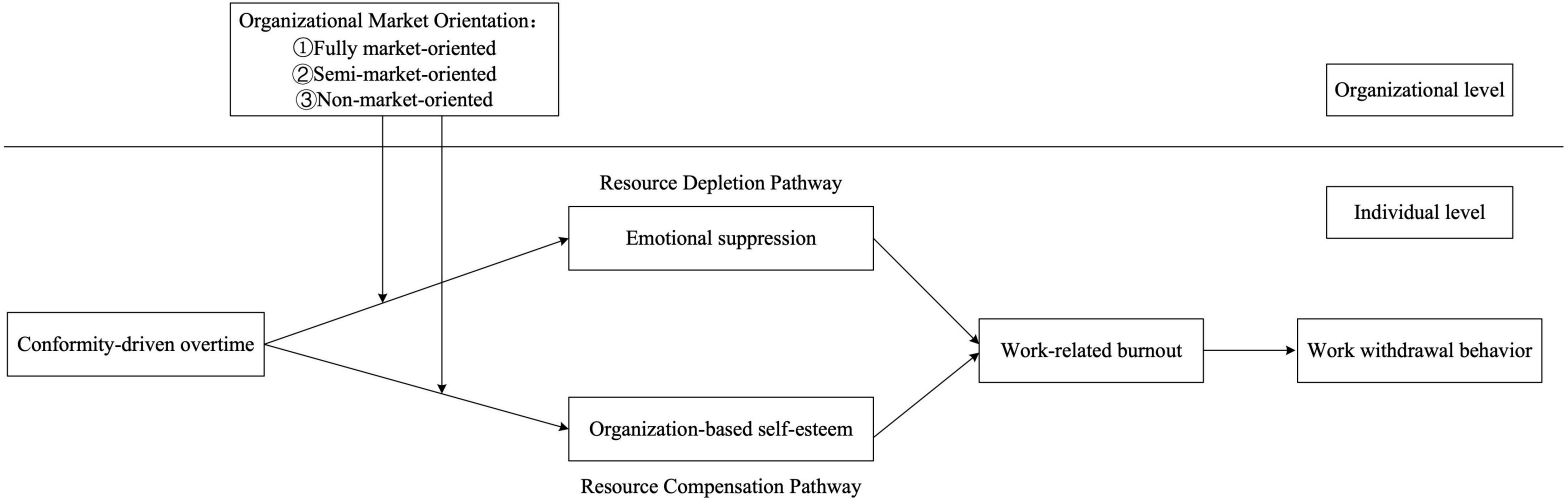
Theoretical model.

Figure 1 presents the theoretical model of this study.

## 3 Sample and research procedure

### 3.1 Research sample and data collection process

This study employed a four-wave longitudinal survey design with 1-week intervals between waves to minimize common method bias and reverse causality concerns (Johnson et al., 2011; Podsakoff et al., 2012), collecting data via the Wenjuanxing online platform, which prior research confirms yields valid and reliable data comparable to other convenience sampling methods (Lin et al., 2022; Peer et al., 2017; Sherf and Morrison, 2020). Wave 1 (T1) collected demographics, CDO, and organizational market orientation type (*n* = 1,050); Wave 2 (T2) measured ES and OBSE (*n* = 1,021, attrition = 2.76%); Wave 3 (T3) assessed WRB (*n* = 986, attrition = 3.43%); and Wave 4 (T4) captured WWB and performance appraisal fairness (*n* = 943, attrition = 4.36%), with an acceptable total attrition of 10.19%.

To assess whether attrition was random, we conducted a comprehensive attrition analysis. First and most critically, independent samples *t*-tests comparing the final sample (*N* = 943) with those who dropped out (*N* = 107) revealed no significant differences on key demographic variables (e.g., gender, age, organizational characteristics) or substantive variables at Time 1 (e.g., CDO, *p* > 0.05). Second, attrition rates did not differ significantly across organizational types [χ^2^ (2) = 1.85, *p* = 0.40]: 9.8% in fully market-oriented, 10.1% in semi-market-oriented, and 10.7% in non-market-oriented organizations. Third, temporal analysis indicated that attrition was evenly distributed (all wave-to-wave rates < 5%) and survey completion time at Time 1 was not correlated with dropout (*r* = -0.04, *p* = 0.29), suggesting that baseline work intensity did not predict attrition. These convergent findings support the assumption that data were Missing Completely at Random (MCAR) and minimize concerns regarding selection bias.

Accordingly, to ensure data integrity for the longitudinal analysis, we handled missing data using listwise deletion, excluding participants who failed to complete all four waves. This approach is deemed appropriate and rigorous given that: (1) the overall attrition rate was low (10.19%); and (2) the established randomness of the attrition ensures that excluding incomplete cases does not introduce systematic bias into the structural equation modeling estimates.

The final sample of 943 participants demonstrated strong representativeness: 52.81% female, 85.26% aged 26–45, highly educated (47.61% bachelor’s, 44.22% master’s degrees), diverse occupational distribution (39.34% professional/technical, 33.40% production/service, 14.95% management, 12.30% administrative), balanced organizational types (40.51% fully market-oriented, 31.60% semi-market-oriented, 27.89% non-market-oriented), predominantly from state-owned (52.60%) and private enterprises (25.66%), with 93.11% full-time employees, effectively capturing the heterogeneity of Chinese workplace contexts.

The 943 participants were nested within 96 organizations. To justify the use of multilevel modeling, we calculated the Intraclass Correlation Coefficient (1) values for the focal variables using null models: OBSE (0.24), CDO (0.19), WRB (0.16), ES (0.14), and WWB (0.13). All values exceeded the common threshold of 0.12, warranting the use of multilevel analysis to account for organizational clustering.

### 3.2 Measures

All measurement scales implemented were originally developed in English. To ensure measurement equivalence for the Chinese sample, this study followed the standard translation-back-translation procedure recommended by Brislin (1970). First, two bilingual researchers specializing in human resource management independently translated all items from English to Chinese, with discrepancies resolved through discussion to establish a preliminary Chinese version. Second, a third bilingual researcher in the field of organizational behavior, blind to the original English version, back-translated the Chinese items into English. Third, the back-translated version was compared with the original scales to identify semantic inconsistencies, minor adjustments were made to ensure conceptual equivalence while preserving linguistic naturalness. Finally, a panel of three researchers reviewed the finalized Chinese items to verify their readability and cultural appropriateness, confirming that the instruments accurately captured the intended constructs in the Chinese context.

#### 3.2.1 Conformity-driven overtime

CDO was measured with five items developed by Watanabe and Yamauchi (2018). This construct was defined as overtime generated from implicit pressures from colleagues/supervisors, where workplace norms require staying when other members are working. Items assessed the extent to which nurses felt pressured to conform to group expectations regarding overtime work (e.g., “I feel uneasy to leave, even when my work is done, out of regard to my colleagues,” “I feel uneasy to go back when my boss and superiors still remain in my ward”). Items were rated on a 5-point scale (1 = strongly disagree to 5 = strongly agree). The structure of this measure was previously validated through exploratory factor analysis (Watanabe, 2017).

#### 3.2.2 Emotional suppression

ES (α = 0.73) was measured with four items from the Emotion Regulation Questionnaire (ERQ) developed by Gross and John (2003). This construct was defined as a form of response modulation that involves inhibiting ongoing emotion-expressive behavior. Items assessed the extent to which individuals suppress the behavioral expression of both positive and negative emotions (e.g., “I control my emotions by not expressing them,” “When I am feeling negative emotions, I make sure not to express them,” “I keep my emotions to myself”). Items were rated on a 7-point scale (1 = strongly disagree to 7 = strongly agree). We employed the complete 4-item ERQ suppression subscale, encompassing items for both positive and negative emotion suppression, rather than focusing exclusively on negative emotion items. This decision was based on three considerations. First, the ERQ suppression subscale was validated as a unidimensional measure capturing general expressive suppression rather than valence-specific suppression (Gross and John, 2003). Factor analytic studies demonstrate that suppression operates as a unified regulatory strategy. Second, emotion regulation research shows that suppressing negative emotions in response to stressors generalizes to overall emotional expressiveness, including reduced positive emotional expression (Gross, 2002; Butler et al., 2003). The regulatory resources required to suppress frustration or resentment (negative emotions from CDO) deplete the same resources needed for spontaneous positive emotional expression. Third, the scale includes two valence-neutral items (“I control my emotions by not expressing them”; “I keep my emotions to myself”) that already assess general emotional inhibition. Thus, the complete scale appropriately captures the general regulatory process through which CDO depletes emotional resources.

#### 3.2.3 Organization-based self-esteem

OBSE (α = 0.91) was measured with ten items developed by Pierce et al. (1989). This construct was defined as the degree to which organizational members believe that they can satisfy their needs by participating in roles within the context of an organization. Items assessed the extent to which employees perceive themselves as important, meaningful, effectual, and worthwhile within their employing organization (e.g., “I count around here,” “I can make a difference around here”). Items were rated on a 5-point scale (1 = strongly disagree to 5 = strongly agree).

#### 3.2.4 Work-related burnout

WRB (α = 0.87) was measured with seven items from the WRB subscale of the Copenhagen Burnout Inventory (CBI) developed by Kristensen et al. (2005). This construct was defined as the degree of physical and psychological fatigue and exhaustion that is perceived by the person as related to his/her work. The WRB subscale was selected because it has broad applicability across different occupational groups, unlike the client-related burnout subscale which is specific to human service work. Items assessed various aspects of work-related exhaustion and fatigue (e.g., “Do you feel worn out at the end of the working day?,” “Are you exhausted in the morning at the thought of another day at work?,” “Is your work emotionally exhausting?”). Items were rated on a 5-point scale ranging from 1 (never/almost never or to a very low degree) to 5 (always or to a very high degree), with one item reverse-scored.

Work Withdrawal Behavior. WWB was assessed using the Psychological Withdrawal subscale (α = 0.84) from Lehman and Simpson’s (1992) scale. This subscale is grounded in the neglect dimension of Hirschman’s (1970) Exit, Voice, Loyalty, and Neglect (ELVN) typology. The subscale evaluates the extent to which employees psychologically withdraw from the work situation, encompassing behaviors such as: thoughts of being absent, daydreaming, engaging in personal tasks during work hours, excessive chatting with colleagues, exerting minimal effort at work, and delegating one’s own work to others (e.g., “How often have you had thoughts of being absent from work?,” “How often do you daydream at work?,” “How often do you let others do work that you should be doing?”). Items were rated on a 7-point scale where 1 = never/none and 7 = very often/11 or more times, reflecting behavioral frequency over the preceding 12 months.

#### 3.2.5 Organizational market orientation

Organizational market orientation was classified according to the official framework outlined in the “*Guiding Opinions of the Central Committee of the Communist Party of China and the State Council on Deepening the Reform of State-Owned Enterprises” (Zhongfa [2015] No. 22)*. This variable was defined based on the degree of market competition the organizations face and the strength of profit-seeking imperatives embedded in their operational logic. Participants were asked to classify their organization into one of three categories based on its level of market orientation: (1) Fully market-oriented organizations—organizations that operate in highly competitive markets, prioritize profitability as their primary objective, face substantial performance pressures, and typically link compensation closely to individual or organizational performance (e.g., private enterprises and state-owned enterprises that fully exposed to market competition); (2) Semi-market-oriented organizations—organizations that operate in sectors with state priority support or certain monopolistic advantages, that are subject to both profitability pressures and policy-mandated responsibilities, experience limited market competition, and often benefit from policy protection or preferential access to state resources (e.g., strategic state-owned enterprises); (3) Non-market-oriented organizations—organizations that do not primarily pursue profitability, provide essential public or social services, rely on relatively stable funding sources, and face minimal performance pressure (e.g., public service organizations and governmental units). To corroborate the validity of our organizational market orientation classification, we employed two complementary validation strategies. First, we conducted an objective verification procedure. Two independent research assistants, blinded to the study hypotheses, retrieved official organizational data (via the National Enterprise Credit Information Publicity System, official websites, and government directories) for a randomly selected subset of participants (*n* = 150, roughly 16% of the sample). They independently classified each organization based on objective criteria—including ownership structure, business domain, and operational mandate—and compared these coding with participants’ self-reports. The analysis revealed a high concordance rate (98.7%; 148 of 150 cases), with the two discrepant cases involving mixed-ownership enterprises characterized by inherently ambiguous structural classifications. Second, we established criterion-related validity by testing whether market orientation predicted overtime frequency, a behavioral proxy for competitive pressure. As theoretically expected, an ANOVA confirmed a distinct gradient: non-market organizations exhibited the lowest frequency (*M* = 1.62), semi-market organizations showed moderate frequency (*M* = 2.28), and fully market-oriented organizations demonstrated the highest frequency [*M* = 3.16), *F* (2, 940) = 271.55, *p* < 0.001, η^2^ = 0.366]. Collectively, these convergent findings provide robust evidence that our classification measure accurately captures the intended structural differences across organizational types.

#### 3.2.6 Control variables

This study controlled for a series of demographic variables, including gender, age group, education level, job type, position level, years of service, and income level (Becker et al., 2016; Beckers et al., 2008). The inclusion of these variables is grounded in solid theoretical foundations and empirical support. We also controlled for work-related variables, specifically overtime frequency, overtime pay, employment status, and position level (Yang et al., 2023; Cherry, 2004; Li and Siegrist, 2018). It should be noted that although organizational ownership (e.g., state-owned enterprises, private enterprises, foreign-invested enterprises) is commonly included as a control variable in organizational behavior research, we did not incorporate it into our control variable system. On one hand, organizational ownership substantially overlaps with our moderator variable—organizational market orientation type—in both conceptual meaning and operational definition. On the other hand, potential endogeneity between these two variables may exist, and forcibly controlling for organizational ownership could mask the true pathways through which the moderating mechanism operates, thereby compromising the explanatory validity of our model.

### 3.3 Descriptive statistics and correlation analysis results

The mean scores for the key variables were as follows: CDO (*M* = 3.01, *SD* = 0.98), ES (*M* = 4.00, *SD* = 1.83), OBSE (*M* = 3.00, *SD* = 0.99), WRB (*M* = 3.01, *SD* = 1.01), WWB (*M* = 4.02, *SD* = 1.82). Regarding the distribution of organizational market orientation, 41% of the sample worked in fully market-oriented organizations and 32% in semi-market-oriented organizations, reflecting an appropriate distribution across different organizational market orientation levels. The correlation analysis results supported the theoretical hypotheses. CDO was significantly and positively correlated with OBSE (*r* = 0.48, *p* < 0.001), ES (*r* = 0.12, *p* < 0.001), WRB (*r* = 0.13, *p* < 0.001), and WWB (*r* = 0.17, *p* < 0.001). Regarding the mediator variables, ES was significantly and positively correlated with WRB (*r* = 0.28, *p* < 0.001), OBSE was significantly and negatively correlated with WRB (*r* = –0.12, *p* < 0.001), and WRB was significantly and positively correlated with WWB (*r* = 0.22, *p* < 0.001).

### 3.4 Reliability, validity tests, and confirmatory factor analysis

To ensure the quality of measurement instruments, we conducted comprehensive reliability and validity tests for all scales. The reliability tests demonstrated excellent internal consistency across all measures. Specifically, the Cronbach’s α coefficients were as follows: CDO (α = 0.956), ES (α = 0.947), OBSE (α = 0.972), WRB (α = 0.965), WWB (α = 0.972). All these values far exceeded the 0.9 threshold for excellent reliability, indicating strong internal consistency for all scales.

Regarding convergent validity, we assessed each construct using average variance extracted (AVE). The results showed AVE values of 0.815 for CDO, 0.818 for ES, 0.778 for OBSE, 0.797 for WRB, 0.816 for WWB. All values exceeded the recommended threshold of 0.5, indicating that the measurement items adequately captured the intended latent constructs. Furthermore, comparison of AVE values with inter-construct correlations revealed that all AVE values were higher than their corresponding correlation coefficients with other constructs. For instance, the highest correlation was between CDO and OBSE (*r* = 0.48), which was still significantly lower than the AVE values for CDO (AVE = 0.815) and OBSE (AVE = 0.778), demonstrating good discriminant validity.

To further verify discriminant validity among constructs, we conducted confirmatory factor analysis (CFA) to test the theoretical model, with results presented in Table 2. The hypothesized five-factor model (treating CDO, ES, OBSE, WRB, and WWB as five distinct factors) demonstrated excellent fit: χ^2^(517) = 463.22, CFI = 1.00, TLI = 1.00, RMSEA = 0.00, SRMR (within) = 0.02. All indices met the ideal criteria for model fit, confirming the appropriateness of the five-factor structure. To test discriminant validity more rigorously, we constructed five competing four-factor models for comparison. These models combined theoretically related constructs that might potentially overlap. All four-factor models showed significantly poorer fit than the five-factor model. For example, the model combining CDO with ES showed deteriorated fit with χ^2^ increasing to 4115.30, CFI decreasing to 0.90, and RMSEA increasing to 0.09. The model combining ES with WRB showed similar deterioration (χ^2^ = 3851.77, CFI = 0.90, RMSEA = 0.08). The remaining three competing models performed even worse, with CFI values falling below 0.80 and RMSEA exceeding 0.12. Additionally, the information criteria indicated that the five-factor model had the lowest AIC (78238) and BIC (78781) values, further supporting its superiority.

**TABLE 1:** Descriptive statistics and correlations for study variables.

Variable	M	SD	1	2	3	4	5	6	7	8	9
**Level-1 variables**											
1. Gender	0.47	0.50	–								
2. Age group	2.5	0.78	–0.01	–							
3. Education level	3.56	0.66	–0.03	0.01	–						
4. Position level	1.27	0.59	0.03	–0.01	0.03	–					
5. Tenure	2.53	1.04	0.03	–0.02	–0.03	–0.01	–				
6. Income level	3.12	0.87	0.00	‘0.03	–0.02	0.00	0.03	–			
7. Overtime frequency	2.45	1.05	–0.02	0.02	–0.12***	0.05	0.02	0.11***	–		
8. Overtime pay	0.67	0.47	–0.03	–0.05	–0.11**	0.03	0.03	0.14***	0.45***	–	
9. Employment status	0.93	0.25	0.01	0.00	0.03	–0.03	–0.02	–0.01	–0.08*	–0.07*	–
10. Job type: professional/technical	0.39	0.49	–0.01	–0.02	0.02	–0.02	–0.02	–0.05	–0.13***	–0.15***	0.04
11. Job type: production/service	0.33	0.47	–0.03	–0.03	–0.04	0.03	–0.01	0.09**	0.19***	0.23***	–0.08*
12. Job type: administrative/law enforcement	0.12	0.33	0.01	0.02	–0.03	0.01	0.04	–0.03	0.07*	0.05	0.03
13. Conformity-driven overtime	3.01	0.98	0.04	–0.05	0.00	0.05	–0.01	0.01	0.03	–0.01	0.03
14. Emotional suppression	4.00	1.83	–0.09**	0.02	–0.01	–0.04	0.00	0.04	0.03	0.01	–0.04
15. Organization-based self-esteem	3.00	0.99	0.05	–0.03	0.03	0.04	0.00	0.05	0.07*	0.06	0.00
16. Work-related burnout	3.01	1.01	–0.01	0.02	–0.01	–0.01	0.07*	0.05	0.05	0.02	–0.01
17. Work withdrawal behavior	4.02	1.82	–0.02	–0.02	0.01	–0.06	–0.08*	–0.01	0.02	0.08**	0.00
**Level-2 variables**											
18. Semi-market-oriented	0.32	0.47	0.05	0.04	0.03	–0.03	0.01	0.00	–0.11***	0.28***	0.02
19. Fully market-oriented	0.41	0.49	–0.07*	–0.11**	–0.13***	0.04	0.00	0.16***	0.55***	0.49***	–0.12***
	**M**	**SD**	**10**	**11**	**12**	**13**	**14**	**15**	**16**	**17**	**18**
**Level-1 variables**											
10. Job type: professional/technical	0.39	0.49	–								
11. Job type: production/service	0.33	0.47	–0.57***	–							
12. Job type: administrative/law enforcement	0.12	0.33	–0.30***	–0.27***	–						
13. Conformity-driven overtime	3.00	0.98	–0.03	–0.01	–0.02	–					
14. Emotional suppression	4.00	1.83	–0.05	0.00	0.04	0.12***	–				
15. Organization-based self-esteem	3.00	0.99	–0.02	0.00	–0.01	0.48***	0.04	–			
16. Work-related burnout	3.01	1.01	0.01	0.02	–0.01	0.13***	0.28***	–0.12***	–		
17. Work withdrawal behavior	4.02	1.82	0.01	0.00	–0.01	0.17***	0.08*	0.01	0.22***	–	
**Level-2 variables**											
18. Semi-market-oriented	0.32	0.47	0.00	–0.08*	0.15***	0.02	0.00	0.00	–0.02	–0.01	–
19. Fully market-oriented	0.41	0.49	–0.16***	0.31***	–0.07*	–0.01	0.00	0.07*	0.03	0.08*	–0.56***

*p < 0.05,

**p < 0.01,

***p < 0.001.

**TABLE 2:** Confirmatory factor analysis.

Model	χ ^2^	*df*	CFI	TLI	RMSEA	SRMR (within)	AIC	BIC
Five-factor model	463.22	517	1	1	0.00	0.02	78,238	78,781
Four-factor model 1: CDO + ES	4115.30	521	0.90	0.89	0.09	0.09	81,964	82,487
Four-factor model 2: ES + WRB	3851.77	521	0.90	0.90	0.08	0.08	81,808	82,332
Four-factor model 3: OBSE + WRB	7380.49	521	0.80	0.79	0.12	0.16	85,766	86,290
Four-factor model 4: WRB + WWB	9433.18	521	0.74	0.73	0.14	0.16	87,494	88,017
Four-factor model 5: CDO + WWB	9598.54	521	0.74	0.72	0.13	0.17	87,636	88,159

CDO, conformity-driven overtime; ES, emotional suppression; OBSE, organization-based self-esteem; WRB, work-related burnout; WWB, work withdrawal behavior.

### 3.5 Common method bias test

To examine whether common method bias was present in this study, we employed multiple statistical approaches for comprehensive assessment, including Harman’s single-factor test and the unmeasured latent method construct (ULMC) test. The results of Harman’s single-factor test indicated good data suitability and low risk of common method bias. First, the basic suitability tests demonstrated excellent results: the KMO value was 0.961 (excellent level), and Bartlett’s test of sphericity was significant (χ^2^ = 37081.67, *p* < 0.001), indicating that the data were suitable for factor analysis. Principal component analysis revealed five factors with eigenvalues > 1, cumulatively explaining 83.01% of the total variance, which closely aligned with our five-factor theoretical model. More importantly, the first principal component explained only 27.59% of the total variance, well below the stringent 40% threshold. According to the criterion proposed by Podsakoff et al. (2003), this clearly indicates that serious common method bias was not present in the study. The ULMC test further validated the conclusions from Harman’s test. By comparing the five-factor CFA model with the ULMC model that included a method factor, we found no significant difference in model fit between the two models. Specifically, the chi-square difference test showed Δ χ^2^ = 159.915 (Δ *df* = 160, *p* = 0.097 > 0.05), indicating that the addition of the method factor did not significantly improve model fit (Richardson et al., 2009). These results consistently demonstrate that common method bias had minimal impact on our data and does not pose a threat to the main analytical results. The correlation analysis among variables also supported this conclusion, with none of the correlations among core variables exceeding 0.7, thus avoiding multicollinearity issues. Additionally, all factor loadings in the five-factor model exceeded the 0.5 threshold, demonstrating good measurement quality.

## 4 Hypothesis testing

To test the hypotheses, we estimated a structural-equation path (SEM) model with robust standard errors while controlling for gender, age group, education, job type, position level, tenure, income level, overtime frequency, overtime pay, and employment status. Table 2 reports descriptive statistics and bivariate correlations that align with the theorized directions, offering preliminary evidence. Building on this, Table 3 provides formal SEM results and confirms the main paths after controls: the direct path from CDO to WWB is significant (H1 supported; Table 3: β = 0.3191, *p* < 0.001); CDO increases ES (H2a supported; β = 0.2456, *p* < 0.001); ES increases WRB (H2b supported; β = 0.1548, *p* < 0.001); CDO elevates OBSE (OBSE) (H3a supported; β = 0.4773, *p* < 0.001); OBSE reduces WRB (H3b supported; β = -0.1232, *p* < 0.001); and WRB predicts WWB (H4 supported; β = 0.4096, *p* < 0.001). For H2c, the path from CDO to ES is positive and significant (β = 0.2456, *p* < 0.001), and the path from ES to WRB is positive and significant (β = 0.1548, *p* < 0.001); for H3c, the path from CDO to OBSE is positive and significant (β = 0.4773, *p* < 0.001), and the path from OBSE to WRB is negative and significant (β = -0.1232, *p* < 0.001). Together, these coefficients indicate that ES and OBSE mediate the relationship between CDO and WRB as hypothesized.

**TABLE 3:** Structural equation path analysis results without interaction terms.

Variable	Emotional suppression		Organization-based self-esteem		Work-related burnout			Work withdrawal behavior		
(Intercept)	3.4797*** (0.577)	2.6608*** (0.6067)	2.6782*** (0.3138)	1.0867*** (0.2933)	2.5074*** (0.3187)	1.9688*** (0.312)	2.8374*** (0.3287)	4.5644*** (0.5732)	3.5005*** (0.5988)	3.5374*** (0.5767)
Gender	0.3284** (0.1193)	0.3475** (0.1184)	–0.0999 (0.0648)	–0.0626 (0.0572)	0.0197 (0.0659)	–0.0311 (0.0635)	0.0074 (0.0655)	0.0561 (0.1185)	0.0810 (0.1168)	0.0481 (0.1154)
Age group	0.0419 (0.0764)	0.0594 (0.0759)	–0.0443 (0.0416)	–0.0103 (0.0367)	0.0256 (0.0422)	0.0191 (0.0406)	0.0201 (0.042)	–0.0429 (0.0759)	–0.0202 (0.0749)	–0.0534 (0.074)
Education level	–0.0357 (0.0914)	–0.0357 (0.0907)	0.0603 (0.0497)	0.0603 (0.0438)	–0.0101 (0.0505)	–0.0045 (0.0485)	–0.0026 (0.0502)	0.033 (0.0908)	0.0330 (0.0895)	0.0371 (0.0885)
Job type: professional/technical	–0.2526 (0.181)	–0.2052 (0.1799)	–0.1296 (0.0984)	–0.0375 (0.087)	0.0637 (0.1)	0.1028 (0.0961)	0.0477 (0.0994)	0.0006 (0.1798)	0.0622 (0.1776)	–0.0255 (0.1752)
Job type: production/service	–0.1698 (0.1908)	–0.1256 (0.1896)	–0.1469 (0.1038)	–0.061 (0.0916)	0.0512 (0.1054)	0.0775 (0.1013)	0.0331 (0.1048)	–0.1011 (0.1896)	–0.0437 (0.1871)	–0.1221 (0.1847)
Job type: administrative/law enforcement	0.0432 (0.2315)	0.0988 (0.23)	–0.162 (0.1258)	–0.0538 (0.1112)	0.0079 (0.1278)	0.0012 (0.1228)	–0.0121 (0.1271)	–0.1088 (0.2299)	–0.0365 (0.2270)	–0.1121 (0.224)
Position level	–0.1177 (0.1002)	–0.1375 (0.0995)	0.0642 (0.0545)	0.0256 (0.0481)	–0.0138 (0.0554)	0.0044 (0.0532)	–0.0059 (0.055)	–0.1829 (0.0996)	–0.2087* (0.0982)	–0.1772 (0.097)
Tenure	–0.004 (0.0571)	–0.0012 (0.0566)	–0.0069 (0.031)	–0.0015 (0.0274)	0.0631* (0.0315)	0.0637* (0.0303)	0.0622* (0.0313)	–0.1475** (0.0567)	–0.1439* (0.0559)	–0.1734** (0.0553)
Income level	0.0894 (0.0694)	0.0873 (0.0688)	0.0443 (0.0377)	0.0402 (0.0333)	0.0544 (0.0383)	0.0406 (0.0368)	0.0599 (0.0381)	–0.0318 (0.0689)	–0.0346 (0.0679)	–0.0541 (0.0672)
Overtime frequency	0.0335 (0.0645)	0.0206 (0.064)	0.0607 (0.0351)	0.0356 (0.0309)	0.0471 (0.0356)	0.042 (0.0342)	0.0546 (0.0354)	–0.0273 (0.064)	–0.0441 (0.0632)	–0.0466 (0.0624)
Overtime pay	–0.0413 (0.1449)	–0.0274 (0.1438)	0.0828 (0.0788)	0.1097 (0.0695)	–0.0209 (0.08)	–0.0146 (0.0769)	–0.0107 (0.0795)	0.4052** (0.1439)	0.4231 (0.1419)	0.4137** (0.1402)
Employment status	0.3207 (0.2358)	0.3543 (0.234)	–0.0181 (0.1282)	0.0472 (0.1131)	0.0115 (0.1302)	–0.0381 (0.1252)	0.0092 (0.1293)	–0.0359 (0.2342)	0.0078 (0.2309)	–0.0406 (0.2282)
Conformity-driven overtime		0.2456*** (0.0604)		0.4773*** (0.0292)					0.3191*** (0.0596)	
Emotional suppression						0.1548*** (0.0174)				
Organization-based self-esteem							–0.1232*** (0.0331)			
Work-related burnout										0.4096*** (0.0575)
*R* ^2^	0.02	0.03	0.02	0.24	0.01	0.09	0.03	0.02		0.07

*p < 0.05;

**p < 0.01;

***p < 0.001. All coefficients are standardized coefficients; standard errors are shown in parentheses.

Formal indirect-effect tests to WWB using Bootstrap are presented in Table 4. The direct effect from CDO to WWB remains significant [β = 0.201, 95% CI (0.118, 0.285), *p* < 0.001; consistent with Table 3]. The chain mediation via ES → WRB is positive and significant [H5 supported; CDO → ES → WRB → WWB: β = 0.008, 95% CI (0.003, 0.013), *p* < 0.01], whereas the chain mediation via OBSE → WRB is negative and significant [H6 supported; CDO → OBSE → WRB → WWB: β = –0.025, 95% CI (–0.036, –0.014), *p* < 0.001]. Although A4 and A5 are sizable with opposite signs, their sum together with A1–A3 approximately cancels out (A1 + A2 + A3 + A4 + A5 ≈ 0.002), which helps explain why the direct effect remains dominant.

**TABLE 4:** Chain mediation effects test.

Path	Effect	95% CI lower	95% CI upper	Relative mediation effect (%)	Significance
Direct: CDO → WWB	0.201	0.118	0.285	98.93	***
A1: CDO → ES → WWB	0.000	–0.01	0.01	0.12	
A2: CDO → OBSE → WWB	–0.028	–0.067	0.011	–13.91	
A3: CDO → WRB → WWB	0.047	0.026	0.069	23.23	***
A4: CDO → ES → WRB → WWB	0.008	0.003	0.013	3.94	**
A5: CDO → OBSE → WRB → WWB	–0.025	–0.036	–0.014	–12.31	***

*p < 0.05;

**p < 0.01;

***p < 0.001. CDO, Conformity-driven overtime; ES, Emotional suppression; OBSE, Organization-based self-esteem; WRB, Work-related burnout; WWB, Work withdrawal behavior.

The chain mediation effects (ES pathway: β = 0.008; OBSE pathway: β = -0.025) are modest compared to the direct effect (β = 0.201). This pattern reflects three factors: Chain mediation involves multiplicative attenuation across multiple paths, longitudinal designs yield conservative estimates by eliminating common method bias, and these pathways together account for approximately 14% of total effect, consistent with typical mediation findings. Additionally, modest magnitudes may reflect cultural normalization of CDO in Chinese workplaces (Peng, 2020). Despite this, these effects carry practical significance by accumulating across employees and time and identifying specific intervention targets.

**FIGURE 2:**
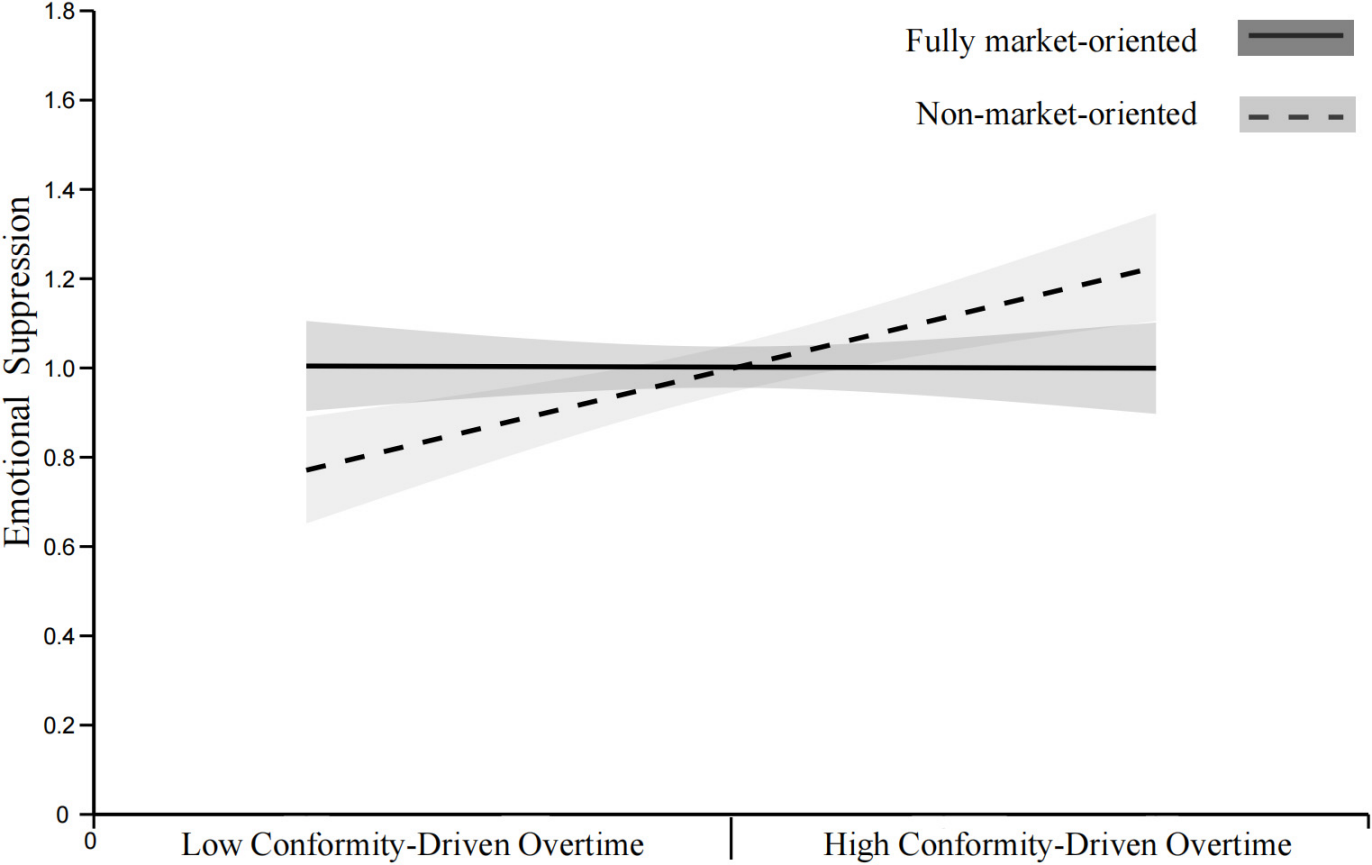
The interactive effect of conformity-driven overtime and organizational market orientation level on emotional suppression (Fully vs. None).

**FIGURE 3:**
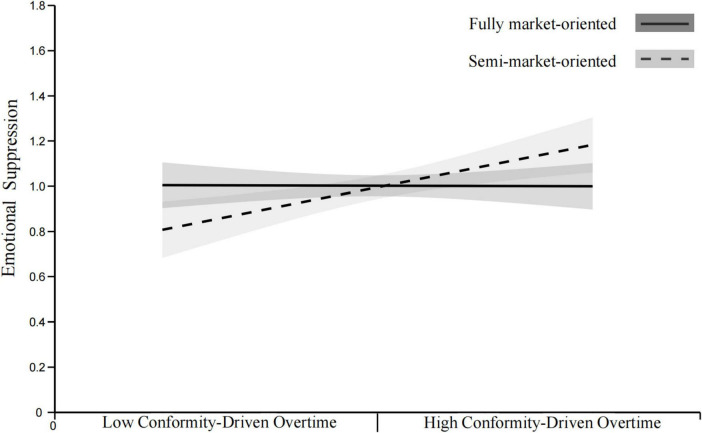
The interactive effect of conformity-driven overtime and organizational market orientation level on emotional suppression (Fully vs. Semi).

**FIGURE 4:**
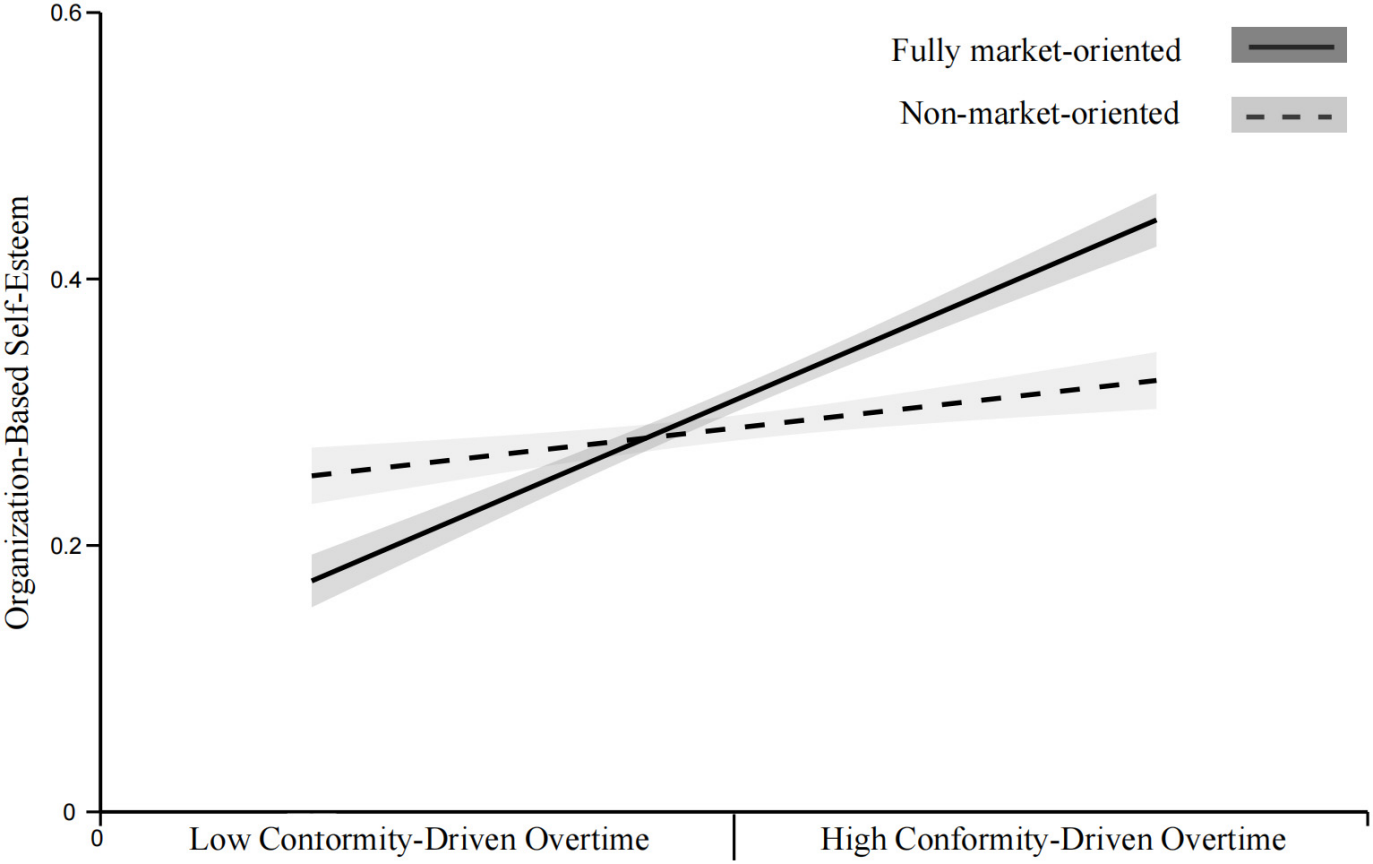
The interactive effect of conformity-driven overtime and organizational market orientation level on organization-based self-esteem (Fully vs. None).

**FIGURE 5:**
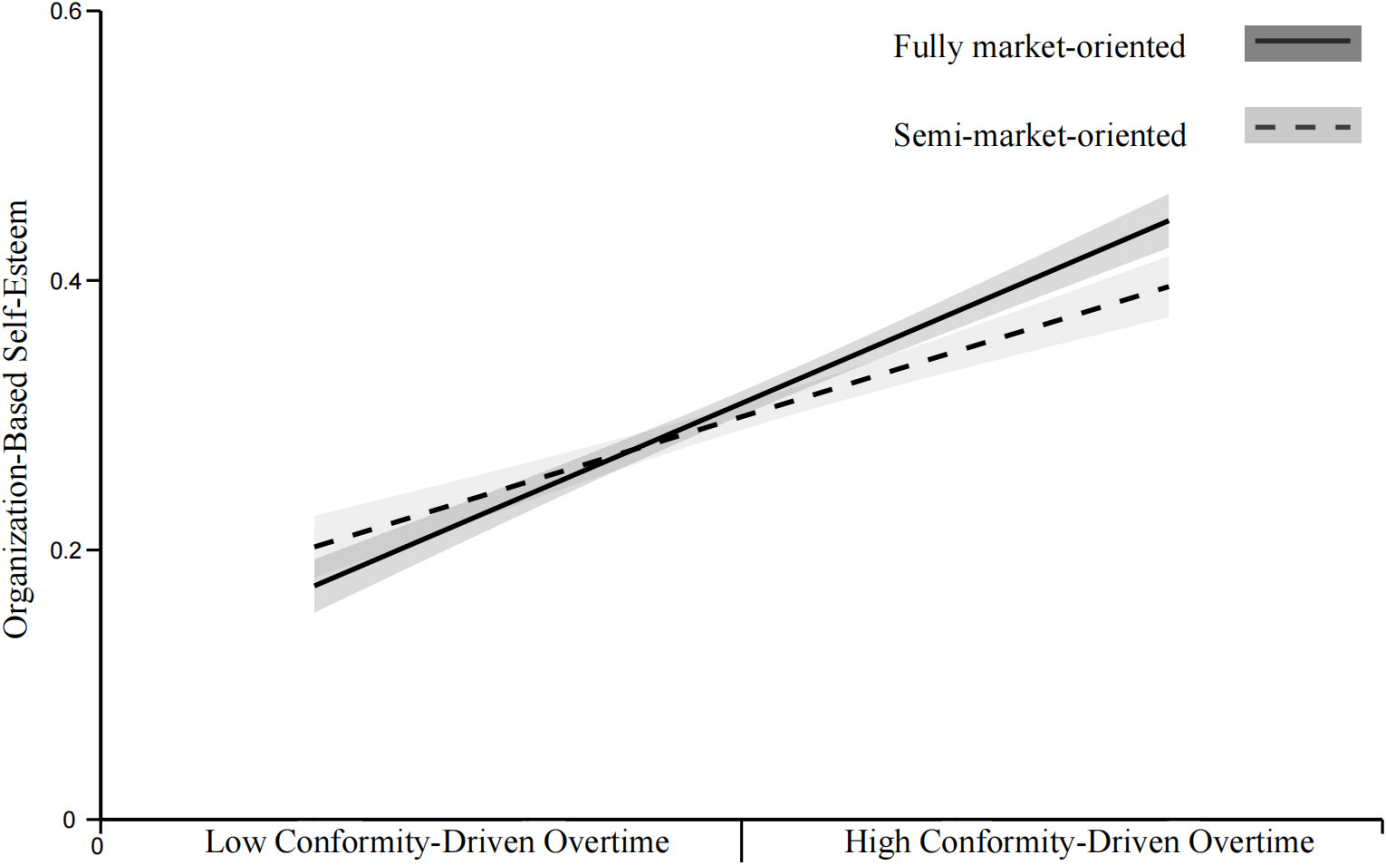
The interactive effect of conformity-driven overtime and organizational market orientation level on organization-based self-esteem (Fully vs. Semi).

**FIGURE 6:**
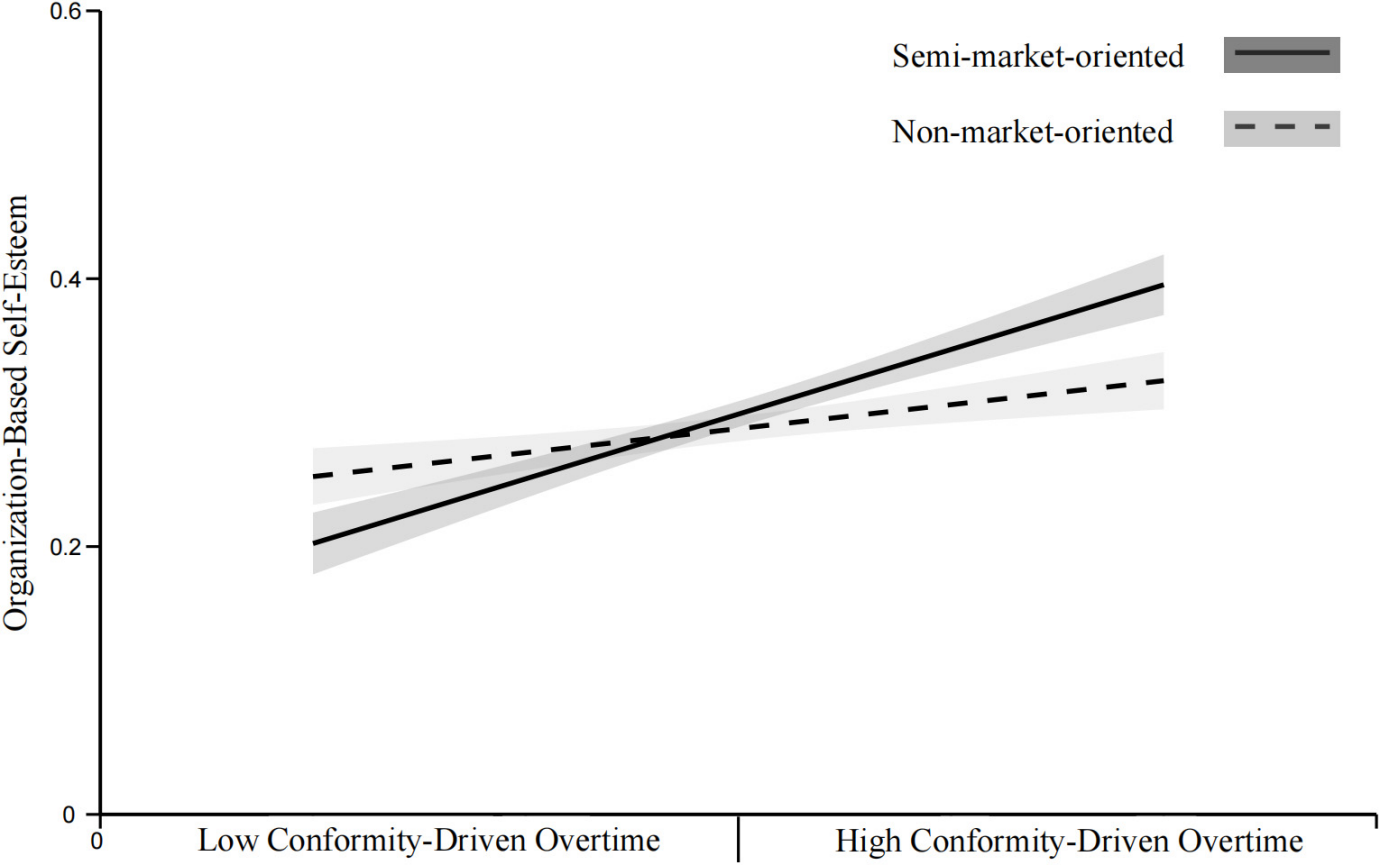
The interactive effect of conformity-driven overtime and organizational market orientation level on organization-based self-esteem (Semi vs. None).

To probe the moderating role of organizational market orientation, we conducted pairwise cross-level interaction analyses across three contrasts—fully vs. non, fully vs. semi, and semi vs. non market-oriented organizations (Table 5): (The interaction term labeled “enterprise_binary.conformity” indexes the moderation within each pairing). Results indicate robust moderation on both pathways from CDO: along the ES path, the moderation effect is significantly negative in fully vs. non (β = -0.543, *p* < 0.001) and fully vs. semi (β = -0.460, *p* < 0.001), but not significant in semi vs. non (β = -0.056, *p* > 0.05), supporting H7a and H7b but not H7c. Along the OBSE path, the moderation effect is significantly positive in all three contrasts—fully vs. non (β = 0.252, *p* < 0.001), fully vs. semi (β = 0.097, *p* < 0.001), and semi vs. non (β = 0.146, *p* < 0.001)—supporting H8a, H8b, and H8c. Collectively, these findings suggest that greater organizational market orientation attenuates the CDO→ES linkage while amplifying the CDO→OBSE linkage, with the strongest effects observed in fully market-oriented firms (see also simple slopes in Figures 2–6). These results provide preliminary evidence of moderation, while tests of moderated mediation require further analysis beyond (Table 5).

**TABLE 5:** Structural equation path analysis results with interaction terms.

Variable	Fully market-oriented vs. Non-market-oriented		Fully market-oriented vs. Semi-market-oriented		Semi-market-oriented vs. Non-market-oriented	
	ES	OBSE	ES	OBSE	ES	OBSE
**Level-1 controls**						
Gender	0.102* (0.045)	0.006 (0.010)	0.049** (0.016)	–0.013 (0.010)	0.091 (0.066)	–0.003 (0.021)
Age group	–0.012 (0.031)	0.003 (0.006)	0.035* (0.018)	–0.003 (0.002)	0.012 (0.049)	0.001 (0.008)
Education level	–0.002 (0.011)	0.007* (0.003)	–0.009 (0.028)	0.006*** (0.001)	–0.030 (0.026)	0.010*** (0.002)
Job type: professional/technical	–0.021 (0.035)	–0.007 (0.012)	–0.082*** (0.016)	0.008* (0.003)	–0.036 (0.054)	–0.009 (0.008)
Job type: production/service	–0.010 (0.040)	–0.014 (0.008)	–0.053*** (0.004)	0.003 (0.003)	–0.004 (0.056)	–0.005 (0.010)
Job type: administrative/law enforcement	0.006** (0.002)	–0.015*** (0.003)	0.020* (0.008)	0.008 (0.012)	0.039*** (0.002)	0.001 (0.015)
Position level	–0.030*** (0.006)	0.001 (0.001)	–0.043*** (0.013)	0.004 (0.004)	–0.043* (0.02)	0.006* (0.003)
Tenure	–0.020 (0.019)	0.001 (0.003)	0.015 (0.020)	0.002 (0.002)	0.000 (0.040)	–0.002** (0.001)
Income level	0.007*** (0.000)	0.004*** (0.001)	0.026 (0.026)	0.004*** (0.001)	0.037 (0.028)	0.003*** (0.001)
Overtime frequency	–0.003 (0.026)	0.001 (0.001)	0.005 (0.031)	0.002*** (0.000)	0.029*** (0.006)	0.001 (0.001)
Overtime pay	0.029 (0.029)	0.018* (0.008)	0.044*** (0.006)	–0.002 (0.016)	0.034* (0.015)	–0.009 (0.007)
Employment status	0.075 (0.087)	0.015 (0.020)	0.127*** (0.010)	0.013 (0.018)	0.038 (0.157)	–0.025* (0.012)
**Level-1 predictors**						
Conformity-driven overtime	0.248*** (0.010)	0.240*** (0.001)	0.233*** (0.005)	0.294*** (0.003)	0.517*** (0.010)	0.165*** (0.000)
**Level-2 predictors**						
Enterprise_binary	–0.035 (0.025)	0.004 (0.012)	–0.002 (0.030)	0.009*** (0.003)	–0.071* (0.031)	0.018*** (0.002)
**Cross-level interactions**						
Enterprise_binary.conformity	–0.543*** (0.001)	0.252*** (0.004)	–0.460*** (0.017)	0.097*** (0.003)	–0.056 (0.041)	0.146*** (0.002)
*R* ^2^	0.043	0.308	0.043	0.329	0.075	0.175

*p < 0.05;

**p < 0.01;

***p < 0.001. All coefficients are standardized coefficients; standard errors are shown in parentheses; ES, emotional suppression; OBSE, organization-based self-esteem.

To further explicate the moderating mechanism of organizational market orientation, we estimated simple slopes of CDO within each organizational market orientation category (see Figures 2–6), consistent with the cross-level interactions reported in Table 5. Along the ES pathway, the slope from CDO to ES was positive and significant in non-market-oriented organizations (β = 0.569, *p* < 0.001), remained significantly positive in semi-market-oriented organizations (β = 0.469, *p* = 0.001), and was essentially null in fully market-oriented organizations (β = -0.006, *p* = 0.957). Along the OBSE pathway, the slope from CDO to OBSE increased monotonically with organizational market orientation—from non-market-oriented organizations (β = 0.089, *p* < 0.001), to semi-market-oriented organizations (β = 0.241, *p* < 0.001), and to fully market-oriented organizations (β = 0.339, *p* < 0.001). Taken together, these simple-slope patterns clarify how organizational market orientation attenuates the CDO → ES linkage while amplifying the CDO → OBSE linkage, providing preliminary evidence of moderation that complements (Table 5) and motivates subsequent tests of moderated mediation.

To examine the moderating role of organizational market orientation on the indirect effects CDO, we conducted group-specific bootstrap analyses with 95% confidence intervals (Table 6).

**TABLE 6:** Indirect effects analysis results.

Path 1: CDO → ES → WRB → WWB				
Moderating variable	Indirect effect	Standard error	95% CI	
			Lower	Upper
Fully market-oriented	–0.008	0.000	–0.009	–0.008
Non-market-oriented	0.007	0.000	0.006	0.007
Difference	–0.015	0.000	–0.015	–0.015
Fully market-oriented	–0.006	0.000	–0.007	–0.006
Semi-market-oriented	0.007	0.000	0.006	0.007
Difference	–0.013	0.000	–0.014	–0.012
Semi-market-oriented	0.011	0.000	0.009	0.013
Non-market-oriented	0.012	0.001	0.012	0.013
Difference	–0.001	0.001	–0.003	0.001
**Path 2: CDO → OBSE → WRB → WWB**				
**Moderating variable**	**Indirect effect**	**Standard error**	**95% CI**	
			**Lower**	**Upper**
Fully market-oriented	–0.047	0.005	–0.058	–0.036
Non-market-oriented	–0.023	0.003	–0.028	–0.018
Difference	–0.024	0.003	–0.03	–0.018
Fully market-oriented	–0.045	0.011	–0.066	–0.024
Semi-market-oriented	–0.034	0.008	–0.05	–0.018
Difference	–0.011	0.003	–0.016	–0.006
Semi-market-oriented	–0.036	0.006	–0.048	–0.024
Non-market-oriented	–0.019	0.003	–0.025	–0.013
Difference	–0.017	0.003	–0.023	–0.011

CDO, Conformity-driven overtime; ES, Emotional suppression; OBSE, Organization-based self-esteem; WRB, Work-related burnout; WWB, Work withdrawal behavior. Organizational market orientation level = Fully market-oriented/Semi-market-oriented/Non-market-oriented.

For the ES pathway (see Path 1 in Table 6), moderated mediation was evident in contrasts involving fully market-oriented organizations: the indirect effect of CDO on WWB through ES was negative in fully market-oriented firms (-0.008) but positive in non-market-oriented firms (0.007), with a significant difference [-0.015, 95% CI (-0.015, -0.015)]. A similar reversal was observed for the fully vs. semi contrast [difference = -0.013, 95% CI (-0.014, -0.012)], whereas the semi vs. non contrast was not significant [difference = -0.001, 95% CI (-0.003, 0.001)]. These findings formally support the moderated mediation hypothesis for ES only in contrasts involving fully market-oriented organizations (H7a and H7b supported).

For the OBSE pathway (see Path 2 in Table 6), moderated mediation exhibited a graded pattern across all three contrasts. The indirect effect consistently reduced WWB in all groups, with the strongest effect in fully market-oriented firms (-0.047) and the weakest effect in non-market-oriented firms (-0.023). All pairwise differences were significant [fully vs. non: -0.024, 95% CI (-0.030, -0.018); fully vs. semi: -0.011, 95% CI (-0.016, -0.006); semi vs. non: -0.017, 95% CI (-0.023, -0.011)]. These results formally support moderated mediation across all contrasts for the OBSE pathway (H8a, H8b, and H8c supported).

## 5 Discussion

Our empirical findings on whether and how CDO leads to WWB reveal a fundamental paradox: CDO operates as a psychologically ambivalent job demand, as employees simultaneously experience resource depletion via ES and resource enhancement via OBSE, both converging on WRB as the pivotal mechanism finally driving WWB. Crucially, organizational market orientation emerged as a boundary condition fundamentally altering these pathways: In fully market-oriented organizations, the ES pathway became non-significant, whereas the OBSE pathway significantly strengthened, suggesting that organizational market orientation transforms not only the strength of these pathways but also the underlying psychological processes shaping how employees experience and respond to CDO.

### 5.1 Theoretical contributions

First and most fundamentally, we reconceptualize overtime, often treated as a uniform stressor (Dembe et al., 2005; Jiang and Yang, 2025), as a psychologically multifaceted phenomenon by introducing and empirically examining CDO as a specific form of overtime work that produces both resource-depleting and resource-enhancing effects. Unlike prior research that treats overtime solely as a quantitative demand and examines its impact primarily in terms of duration or frequency (Jiang and Yang, 2025; Shen et al., 2025; Yang et al., 2025), we demonstrate that the underlying driven factors of overtime (e.g., conformity), as a form of qualitative demand, fundamentally alter the psychological meaning and consequences resulted by overtime work, which cannot be captured by quantitative measures alone. Moreover, by proposing CDO as a formal construct and specifying its conceptual boundaries, we move beyond descriptive accounts toward a theoretically grounded framework for understanding this phenomenon. By doing so, it responds directly to Demerouti’s (2025) call to develop contextual models within JD-R frameworks. Also, this reconceptualization of overtime work challenges the implicit assumption in the overtime literature that “more is worse” (Angrave and Charlwood, 2015; Smith et al., 1998), revealing instead that the same overtime behavior can have fundamentally different implications depending on its underlying driving factors.

Second, we extend JD-R model by empirically validating how a single job demand simultaneously operates through opposing mechanisms—CDO triggers both resource depletion (ES) and compensation (OBSE), providing evidence for “psychological ambivalence” where job demands generate parallel rather than sequential resource-depleting and resource-generating processes. This finding directly responds to Bakker’s et al. (2023) proposition that job demands can yield both positive and negative outcomes but goes further by specifying the precise conditions and mechanisms through which this occurs: when conformity carries social recognition signals, it activates compensatory mechanisms through enhanced OBSE that partially offset the depleting effects of such overtime demands.

Our third contribution is to identify and validate organizational market orientation as an organization-level moderator that is contextually distinctive and objectively measurable in the Chinese setting, directly responding to calls for greater contextualization in human resource management research (Kim and Wright, 2011; Sheldon and Sanders, 2016). Beyond serving as just another moderator, it functions as a theoretical bridge linking macro-institutional structures with micro-psychological processes. Although operationalized in our study as organizational market orientation, we theorize that its influence arises through the clarity and objectivity of overtime evaluation systems, which shape employees’ perceived control over effort–reward contingencies (Hartmann and Slapničar, 2012), thereby altering the effects of CDO. Our findings demonstrate that institutional context is not merely a backdrop but an active force shaping how employees experience job demands, offering rare empirical support for institutional theory’s claim that macro-level logics can reconfigure micro-level psychological processes (Bitektine and Haack, 2015). In doing so, this study also extends the multi-level logic of JD–R model by showing that organizational contexts such as HR practices and organizational climate significantly influence how employees appraise demands and resources (Bakker and Demerouti, 2018; Demerouti, 2025).

### 5.2 Practical implications

Although our study reveals that CDO may yield positive outcomes, specifically enhanced OBSE, it ultimately precipitates WWB. This underscores the critical need for HR managers to discern the underlying drivers of overtime behavior. Specifically, organizations must cultivate a culture where overtime is driven by task necessity rather than compelled by implicit social compulsion.

However, translating this general principle into practice is not a “one-size-fits-all” endeavor. Critically, our results indicate that the impact of CDO is contingent upon organizational market orientation, necessitating context-specific HR strategies tailored to distinct institutional realities.

In fully market-oriented organizations, where a performance logic dominates, managers should prioritize evaluative transparency. Establishing consistent, quantitative performance metrics ensures that employees’ extra efforts are objectively recognized, thereby strengthening the effort-reward linkage and bolstering perceived control.

Conversely, in non-market-oriented organizations (e.g., public institutions) where objective quantification is often structurally constrained, interventions must focus on directly mitigating normative pressure and ES. First, organizations should formalize overtime management through structural mechanisms, specifically mandatory compensatory leave systems. Given that monetary rewards may be restricted, enforcing “use-it-or-lose-it” leave policies provides tangible acknowledgment of effort while ensuring necessary resource recovery. Second, to intercept the ES pathway, leaders must be trained to de-normalize CDO. In high power-distance contexts, managers should explicitly model healthy work-life boundaries, such as leaving on time, to alleviate the implicit pressure for subordinates to remain present. Third, fostering psychological safety is essential. Establishing safe voice channels allows employees to express workload and overtime concerns without fear of political repercussions, thereby diminishing the need for psychologically costly ES.

Finally, addressing the systemic roots of CDO requires coordinated interventions across multiple levels. At the organizational level, leaders must establish clear work-life boundaries and validate high performance achieved without reliance on excessive hours. This approach helps to destigmatize the refusal of non-essential overtime, ensuring it is not misconstrued as a lack of commitment. At the professional level, industry associations should promulgate standards for sustainable work practices to counteract the normalization of chronic overtime as a standard industry expectation. At the societal level, business schools and professional training programs should integrate frameworks for sustainable performance that critically challenge the deep-seated cultural narrative framing overtime as a definitive marker of virtue or professional dedication.

### 5.3 Limitations and future research suggestions

Three limitations merit consideration.

First, while our four-wave longitudinal design strengthens causal inference by establishing temporal precedence and reducing common method bias through staggered measurement, several design features limit the strength of causal claims. Our 4-week observation window may not capture longer-term dynamics: the dual pathways may operate over different time scales (weeks vs. months vs. years), and cumulative effects or adaptation processes may require extended follow-up to detect. Additionally, reliance on self-reported CDO may not fully capture the subtle, often unconscious ways conformity pressures manifest, and participants may underreport such behaviors due to social desirability bias. Future research could address these limitations by incorporating longer observation periods, experience sampling methods to capture within-person fluctuations, and multi-source data (e.g., supervisor ratings of CDO, objective work hour logs) to triangulate self-reports.

Second, sample representativeness raises generalizability concerns. While our sample includes diverse occupations and organizational types, state-owned enterprises (52.60%) and highly educated knowledge workers (91.83% bachelor’s degree or higher) are overrepresented. This distribution reflects our deliberate focus on contexts where CDO is theoretically most salient, yet it limits generalizability to underrepresented groups. For instance, the dynamics may differ significantly for blue-collar workers. Unlike knowledge workers subject to implicit norms, blue-collar workers may experience overtime primarily as task-driven or economically incentivized rather than norm-based. Consequently, the salience of ES as a mediator might be reduced in these contexts, as the psychological conflict of facades is less relevant. Similarly, regarding organizational type, the findings may not fully apply to employees in small private firms. In contrast to the ritualistic conformity often observed in large, bureaucratized SOEs or public institutions, overtime in small firms is likely driven by explicit survival imperatives and direct performance pressure. Thus, the status-signaling function of CDO (enhancing OBSE) may be less pronounced in environments where tangible output is prioritized over visible presence.

Third, the study’s specific institutional and cultural context warrants careful consideration regarding cross-national generalizability. As our findings are derived exclusively from the Chinese setting, unique features such as deep-rooted Confucian values and the specific regulatory environment of Chinese SOEs may not fully translate to other contexts. Therefore, the boundary conditions of our dual-pathway model need to be established across diverse regulatory regimes and cultural values.

To extend these findings, future research should address four specific questions derived from our model.

First, regarding temporal dynamics: Do the dual pathways operate over different time scales? Drawing on Conservation of Resources theory, we hypothesize that the resource-generating effect of OBSE may predominate in the short term (a “honeymoon phase”), whereas the resource-depleting effect of ES likely intensifies over the long term as regulatory resources become exhausted. Longitudinal designs are needed to identify the “tipping point” where CDO transitions from a net gain to a net loss.

Second, regarding individual differences: Do dispositional traits moderate susceptibility? Future studies should examine whether employees high in dispositional conformity experience weaker ES due to better person-environment fit, and whether those with low trait self-esteem derive stronger compensatory OBSE benefits from overtime validation.

Third, regarding practical interventions: Can organizational programs mitigate the negative pathway? We call for field experiments testing whether norm-based interventions—such as correcting “pluralistic ignorance” regarding peer expectations—can reduce the normative pressure driving ES without undermining social cohesion.

Finally, regarding cross-cultural generalizability: How do macro-level cultural and institutional differences moderate these pathways? Culturally, we hypothesize that the compensatory effect (via OBSE) may be attenuated in individualistic Western societies where conformity is often devalued as a lack of autonomy, whereas the depleting effect (via ES) may be intensified due to a stronger conflict between personal authenticity and social pressure. Institutionally, in countries with strict overtime laws (e.g., France or Germany), CDO might be perceived not as a “normative sacrifice” but as an “illegitimate demand” violating legal boundaries, potentially exacerbating strain while diminishing social validation.

## Data Availability

The raw data supporting the conclusions of this article will be made available by the authors, without undue reservation.
